# Variation in social media sensitivity across people and contexts

**DOI:** 10.1038/s41598-024-55064-y

**Published:** 2024-03-19

**Authors:** Sumer S. Vaid, Lara Kroencke, Mahnaz Roshanaei, Sanaz Talaifar, Jeffrey T. Hancock, Mitja D. Back, Samuel D. Gosling, Nilam Ram, Gabriella M. Harari

**Affiliations:** 1 Department of Communication, Stanford University, 450 Jane Stanford Way, Stanford, CA 94305 USA; 2 University of Münster, Münster, Germany; 3 Imperial College London, London, UK; 4 University of Texas at Austin, Austin, USA; 5 Melbourne University, Melbourne, Australia; 6 Present Address: Negotiations, Organizations and Marketing Unit, Harvard Business School, Boston, USA

**Keywords:** Social media, Wellbeing, Physical context, Social context, Personality, Psychology, Human behaviour

## Abstract

Social media impacts people’s wellbeing in different ways, but relatively little is known about why this is the case. Here we introduce the construct of “social media sensitivity” to understand how social media and wellbeing associations differ across people and the contexts in which these platforms are used. In a month-long large-scale intensive longitudinal study (total n = 1632; total number of observations = 120,599), we examined for whom and under which circumstances social media was associated with positive and negative changes in social and affective wellbeing. Applying a combination of frequentist and Bayesian multilevel models, we found a small negative average association between social media use AND subsequent wellbeing, but the associations were heterogenous across people. People with psychologically vulnerable dispositions (e.g., those who were depressed, lonely, not satisfied with life) tended to experience heightened negative social media sensitivity in comparison to people who were not psychologically vulnerable. People also experienced heightened negative social media sensitivity when in certain types of places (e.g., in social places, in nature) and while around certain types of people (e.g., around family members, close ties), as compared to using social media in other contexts. Our results suggest that an understanding of the effects of social media on wellbeing should account for the psychological dispositions of social media users, and the physical and social contexts surrounding their use. We discuss theoretical and practical implications of social media sensitivity for scholars, policymakers, and those in the technology industry.

## Introduction

How does social media impact wellbeing? This is an important question for a variety of stakeholders, ranging from social-media users and academic researchers to those in technology companies and policy makers. Here, we introduce the construct of “social media sensitivity” to help explain how social media’s effects on wellbeing may differ between people (e.g., based on their psychological dispositions) and within a person over time (e.g., based on the context in which the media use is occurring). We conceptualize social media sensitivity as a person-level construct that captures heterogeneity in how individuals respond psychologically to social media within a defined time period (e.g., change after an hour, a day, a week). At its core, social media sensitivity can thus be understood as a state that reflects positive or negative change in a person’s psychological experiences after they have used social media. However, like other constructs, social media sensitivity states can also be aggregated or assessed in reference to longer time periods (e.g., months, years) to reflect a trait-like tendency to respond to social media. As such, people can experience social media sensitivity that is negative (e.g., feeling worse after using social media), positive (e.g., feeling better after using social media), or neutral (e.g., feeling no better or worse after using social media) in the moment, and on average over longer periods of time.

Here, we investigate the psychological and contextual factors that may help explain social media sensitivity at both the between and within-person levels. Using data from two large-scale samples of month-long experience sampling studies, we show that people’s momentary social media sensitivity depends on their psychological dispositions (*who* they are) as well as the physical and social contexts in which they are using social media platforms (*where* and *around whom social media* use is occurring).

Studies from a variety of disciplines have focused on identifying the magnitude and valence of the associations between social media and wellbeing^1^, consistently finding that on average, there is a small and negative association. For example, correlational has generally found small negative associations between social media use and cognitive and social wellbeing^2,3^. Quasi-experimental evidence based on observational data suggests that the introduction of Facebook on college campuses led to small decreases in mental health indices and increases in depression symptomatology^4^. Causal evidence has shown that decreased social media use improves subjective wellbeing [e.g., satisfaction with life, feelings of happiness, loneliness:^3–5^]. Past research suggests that in general, there is a small and negative effect of social media use on wellbeing outcomes, but a growing body of evidence has found there are considerable differences in sensitivity to social media across people, based on who is using these platforms and how they are using them [e.g.,^6,7^]. Moreover, many studies have relied on measuring the associations between social media use and well-being at a single point in time^1,8–10^, precluding the possibility of separating out between-person associations from within-person associations [e.g.,^11^]. As a result, research in this area has generated questions about what drives the heterogeneity of these associations across different people [e.g.,^12^].

The last two decades have witnessed the transformation of social media platforms’ userbase from a relatively small and homogenous group of tech-savvy enthusiasts to include millions of young adults, who have varying psychological dispositions that may make them particularly sensitive to the effects of social media use. The expansion of social media’s user base is consequential for understanding how platforms affect a range of outcomes across wellbeing domains (e.g., affective, social). Hence, one possible explanation for the heterogeneity in social media effects focuses on psychological dispositions to understand *who* is using social media, and whether some people are more psychologically vulnerable than others to the effects of social media use on wellbeing.

Indeed, research suggests that people’s personality traits (e.g., extraversion) are systematically linked with their patterns of social media use^13^, and that psychological dispositions are closely linked to feelings of wellness^14^. In particular, much of the past work has focused on whether people’s dispositional wellbeing, such as their self-esteem^15^, loneliness^16^, and attachment style^17^, explains the relationship between social media use and wellbeing. Past research on this topic suggests that people who are psychologically vulnerable (i.e., those who have a dispositional tendency towards worse wellbeing) tend to suffer greater declines in wellbeing outcomes after using social media, as compared to people who have better dispositional wellbeing. For example, people who are higher in dispositional loneliness tend to feel lonelier after using social media, compared with people who are lower in dispositional loneliness [e.g.,^18^]. The converse is true as well for those who have more sociable dispositions: people who have greater goal-driven behavior are less likely to suffer the negative psychological effects of using social media^19^. Hence, people with greater psychological vulnerabilities tend to have negative social media sensitivities whereas people with lesser psychological vulnerabilities may have positive or neutral social media sensitivities.

When the first social media platforms were launched at the turn of the millennium, they were primarily desktop-based websites that were used in a limited number of places by a relatively small number of people. Over the course of the past two decades, revolutions in computing technologies have transformed social media websites into mobile platforms that are used *on-the-go* in the contexts of everyday life [e.g.,^20,21^]*.* Compared to dispositional traits, relatively little research has examined the extent to which a person’s surrounding context shapes social media sensitivity in the moment^22^. Some empirical evidence supports the idea that people’s physical and social contexts (e.g., where they are and who they are with) can complement or interfere with their social media sensitivities. For instance, when using smartphones while engaging in social interactions, people tend to report lower feelings of enjoyment in comparison to engaging in social interactions without using their smartphones^23^. To the best of our knowledge, only one study has investigated the extent to which places moderate the relationship between social media use and wellbeing outcomes. This study found that using Facebook while at home was linked to lower emotional arousal as compared to using Facebook at other places^24^, suggesting that using social media outside of the home may be favorable for wellbeing.

Cumulatively, past research on social media sensitivity has been subject to several key limitations. First, some of this past research focused on time domains that do not capture everyday social media sensitivity [e.g., instead studying trends aggregated over months and years:^3,25^]. Second, past research into how the effect of social media differs from person to person has recruited participants that are not representative of the largest user base of social media platforms—young adults—focusing instead on adolescents [e.g.,^15^]. Third, a large subset of past research has either failed to capture within-person associations our routinely conflated between and within-person associations between social media use and wellbeing, leading to biased assessments of effect sizes [see^25^ for more details]. Fourth, even when between and within-person effects are modelled separately, past studies have only controlled for a small number of variables (e.g., sex and age) that are correlated with both social media use and wellbeing. Typically, modelling-complexity issues are cited for such modeling decisions [e.g.,^18^], but these can lead to an overestimation of the effect sizes and of the heterogeneity associated with social media use and wellbeing constructs. Fifth, the most recent research has operationalized wellbeing through a small (albeit still important) set of constructs [e.g., self-esteem, attachment style:^24,25^] rather than the capturing the full breadth of what it means to feel well. Lastly, and perhaps most importantly, the bulk of past research has ignored the role played by physical and social contexts in modifying the relationship between social media use and wellbeing [e.g.,^15,20^, for an important exception, see:^26^].

In the present research, we build upon the past research in multiple ways. First, we collect intensive longitudinal data from people’s everyday life, instead of relying on data collected at more coarse temporal domains (e.g., once every six months). Second, unlike recent research that has focused on adolescent populations, our target population of interest is young adults in the United States, who form the largest user-base of social media platforms in the country^27^. To the best of our knowledge, our study is significantly larger in terms of sample size and data collection duration, as compared to past studies about the effects of social media on wellbeing in daily life. Third we deploy a mixture of frequentist and Bayesian analytical strategies that allow us to decompose within and between-person components of social media use. These strategies allow us to specify random slopes (i.e., a slope for each person is estimated) for the relationship between social media use and wellbeing outcomes, which allows us to investigate how social media sensitivity differs across people. Fourth, the richness of our data (e.g., the number of observations per participant) allows us to include many control variables (e.g., sex, age, preceding wellbeing states, engagement in multitasking; see Table S41 for more details) without hindering model computation. Finally, in addition to examining person-level heterogeneity, we also examine how social media sensitivity varies across the physical and social contexts in which these platforms are used.

We structure our contributions using three research questions:

RQ 1: What is the relationship between social media use and subsequent wellbeing in daily life?

RQ 2: To what extent does the relationship between social media use and wellbeing depend on people’s psychological dispositions?

RQ 3: To what extent does the relationship between social media use and wellbeing depend on the physical and social context in which use is occurring?

## Results

Our data were collected from a large sample of young adults in the United States (n_participant_ = 1632; n_obs_ = 120,599) using a combination of cross-sectional surveys and four weeks of experience sampling surveys. We analyzed these data using multilevel models to examine the relationship between social media use and momentary affective and social wellbeing, and to determine whether these associations were moderated by people’s psychological dispositions and the context in which they used social media.

We operationalized social media use by collecting data about whether people used social media (“Social Media Use,” defined as a binary variable of use vs. non-use in a given hour) and the degree of their usage if they had used social media (“Duration of Social Media Use,” defined as the duration of social media use in 15-min increments during the hour). For our affective and social wellbeing outcomes, we asked people to report their momentary stress, affect balance, loneliness, and feelings of being accepted. To assess psychological dispositions, we measured people’s Big Five personality traits (i.e., their levels of openness, extraversion, neuroticism, conscientiousness, and agreeableness)^28^ and dispositional measures of social, affective, and cognitive wellbeing (i.e., loneliness, depression, affect balance, satisfaction with life)^29–32^. To assess people’s physical and social context at the time of social media use, we asked people to report the places they had been (e.g., if they were at home, the gym, in nature when they were using social media platforms) and the people they had spent time with in-person (e.g., if they were around close ties, distant ties, family ties).

To begin examining the construct of social media sensitivity, we first conducted an exploratory analysis of data collected in the fall of 2020 from a sample of 920 participants (observations = 73,284). We used the exploratory findings to generate hypotheses that were then pre-registered for a confirmatory analysis of data collected in the spring of 2021 from a second sample of 764 participants (observations = 55, 903). Our anonymized pre-registration, analytical code and data needed to reproduce all analysis can be found on our project’s Open Science Framework page: https://osf.io/xtpvn/.

 In general, most of the exploratory findings observed in the Fall 2020 dataset did not replicate in the 2021 dataset. For RQ1, 4 of 5 pre-registered hypotheses were confirmed. For RQ2, 2 of 9 pre-registered hypotheses were confirmed. For RQ3, 4 of 12 pre-registered hypotheses were confirmed.

We suspect that the lack of replication across the two datasets might be due, in part, to the macro-level differences in the experiences of our participants. Specifically, the first cohort of participants were living off-campus and experiencing the lockdown period of the COVID-19 pandemic, while the second cohort was back on campus and experiencing the lifting of restrictions on daily life activities. Given such macro-level differences across our two datasets, we subsequently performed a mega analysis^33^, by pooling the exploratory and confirmatory datasets together. Our research questions and analytical approach (e.g., exclusion criteria for observations and participants, modeling strategy) did not change from the preregistration for the purposes of the pooled dataset analysis. Combining the datasets in a mega-analysis allowed us to control for sample-specific differences, permitting a more robust analysis of social media sensitivity. Moreover, this approach allowed us to obtain more reliable point estimates given the increased between-person power. Given that we deviated from our pre-registration plan by focusing on the results from a mega-analysis (instead of exploratory and confirmatory sample findings), the findings should be considered exploratory in nature. We point interested readers to the supplemental materials, which contain the exploratory and confirmatory sample findings. In the main manuscript, we present the results of the mega-analysis.

### What is the relationship between social media use and subsequent wellbeing?

### Social media use (vs non-use)

**Figure 1:**
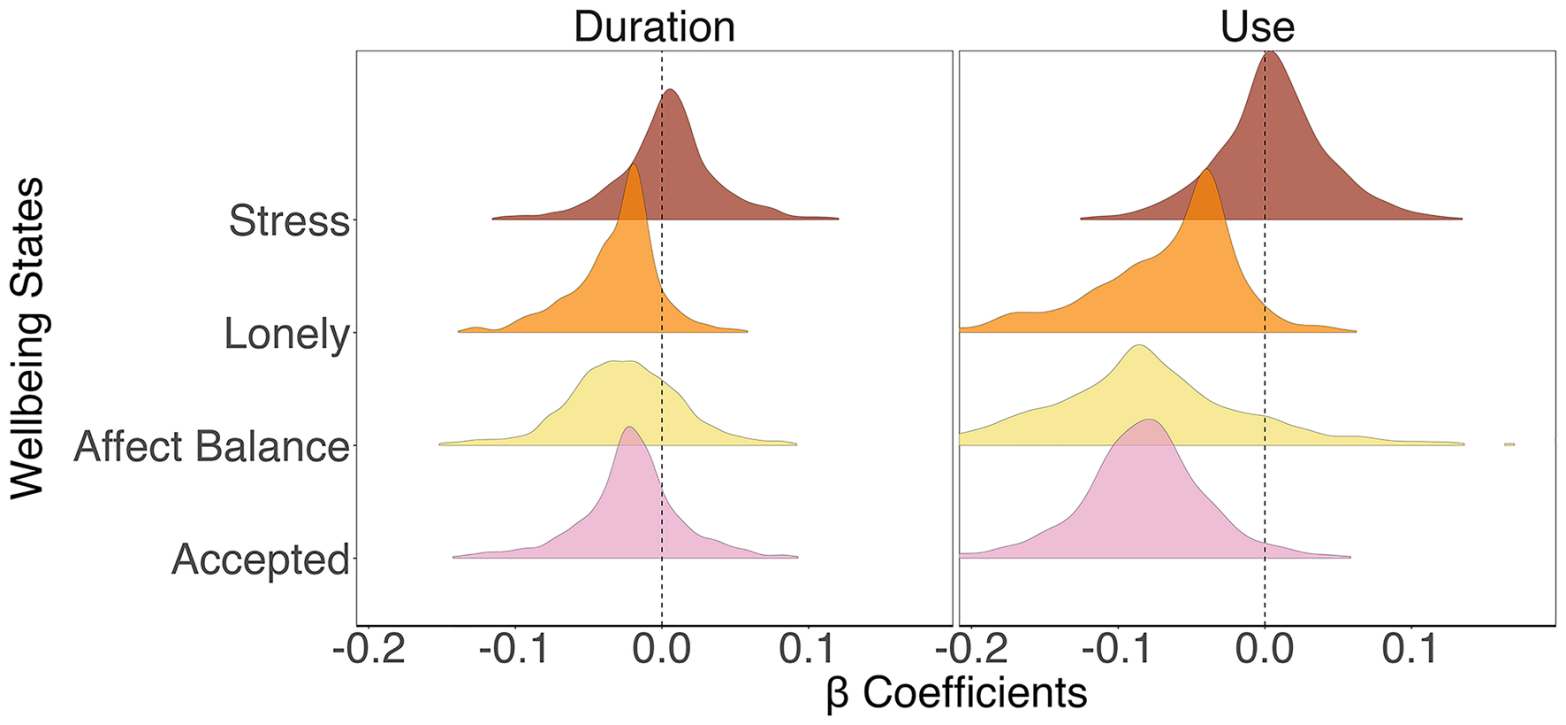
Social media sensitivity across affective and social wellbeing. *Note*: The figure depicts the random-effects coefficients of social media use and wellbeing outcomes. Stress and loneliness were reverse coded such that higher values (> 0) indicate lower levels of stress and loneliness.

People reported lower feelings of being accepted, negative affect balance, and greater feelings of loneliness after using social media, as compared to after not using social media (Fig. 1a).

### Duration of use

People reported negative affect balance, and greater feelings of loneliness after using social media for longer durations than their own average, as compared to after using social media for shorter durations than their own average (Fig. 1b).

### To what extent does the relationship between social media use and wellbeing depend on people's psychological dispositions?

To examine whether between-person differences in psychological dispositions explain the within-person relationship between social media use and momentary wellbeing outcomes, we focus here on the significant cross-level interactions observed. Generally, we find that people who are more psychologically vulnerable feel worse after using social media as compared to people who are less psychologically vulnerable. (There were several significant findings at the between-person level (see supplementary materials). For the purposes of brevity and clarity we have chosen to only interpret the within-person findings in the main text.

#### Loneliness

**Figure 2:**
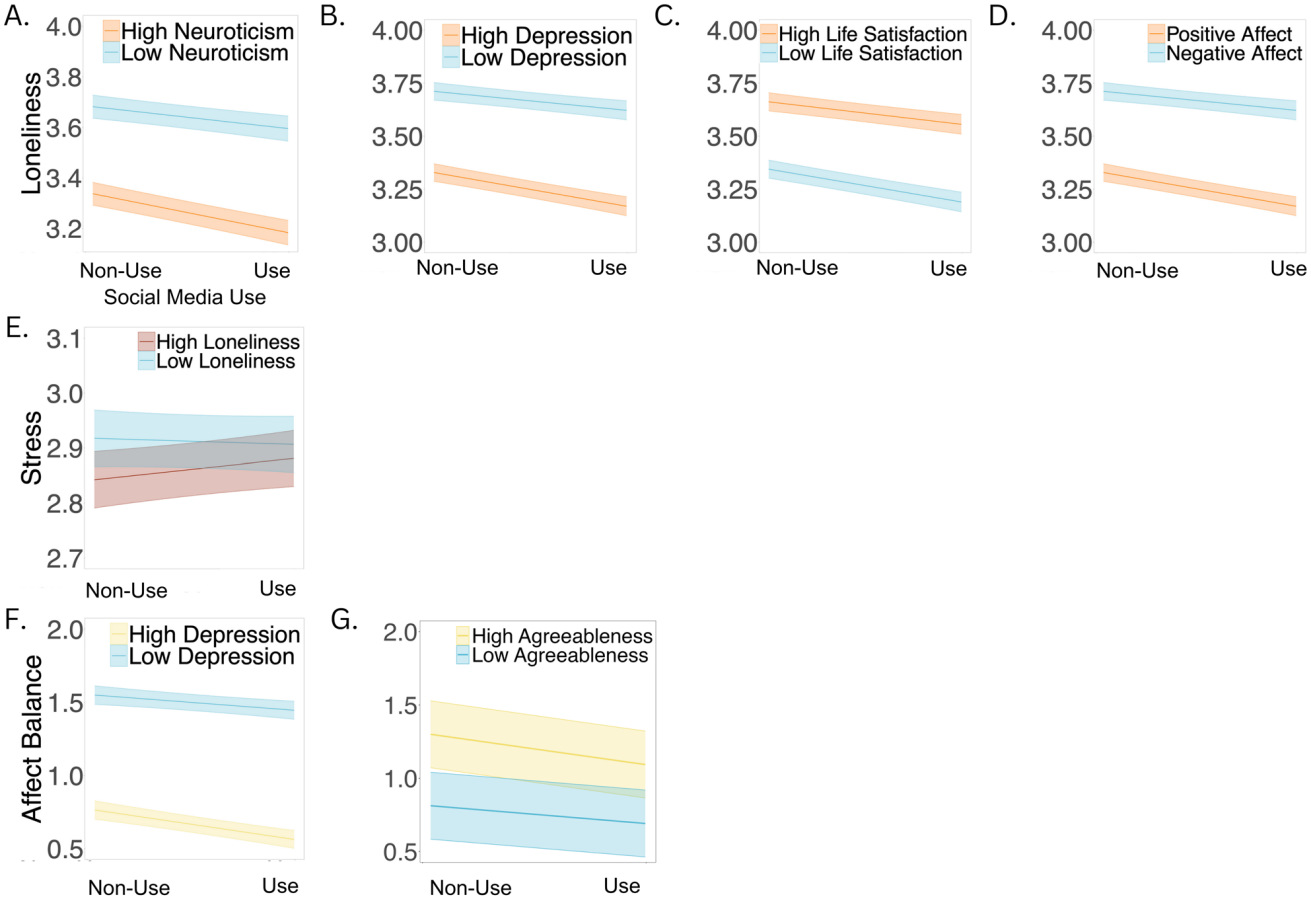
Dispositional moderators. *Note*: Stress and loneliness were reverse coded such that higher values indicate lower levels of stress and loneliness. Bands depict 95% confidence interval of simple slope estimates.

People who were higher in neuroticism (Table S33, Fig. 2a) and depression (Table S33, Fig. 2b) reported feeling lonelier in the moments after using social media platforms, as compared to after not using social media. Similarly, people who were lower in satisfaction with life (Table S33, Fig. 2c) and had a generally negative affect balance (Table S33, Fig. 2d) reported feeling lonelier in the moments after using social media platforms, as compared to after not using social media. In contrast, people who were not psychologically vulnerable (those who were low in neuroticism and depression or had a generally positive affect balance and high satisfaction with life) did not report significant changes in their feelings of loneliness after using social media as compared to not using social media*.*

#### Stress

People who were higher in dispositional loneliness (Table S34 Fig. 2e) reported feeling significantly more stressed after using social media, as compared to people who were lower in dispositional loneliness.

#### Affect balance

People who were higher in depression (Table S35, Fig. 2f) reported a greater negative affect balance in the moments after using social media, as compared to after not using social media. People who were lower in depression did not report significant changes in their affect balance in the moments after using social media. Similarly, people who were higher in agreeableness reported a greater negative affect balance in the moments after using social media, as compared to after not using social media. People who were lower in agreeableness did not report feeling significantly better or worse after using social media (Table S35, Fig. 2g).

### To what extent does the relationship between social media and wellbeing depend on the physical and social context in which social media is being used?

**Figure 3:**
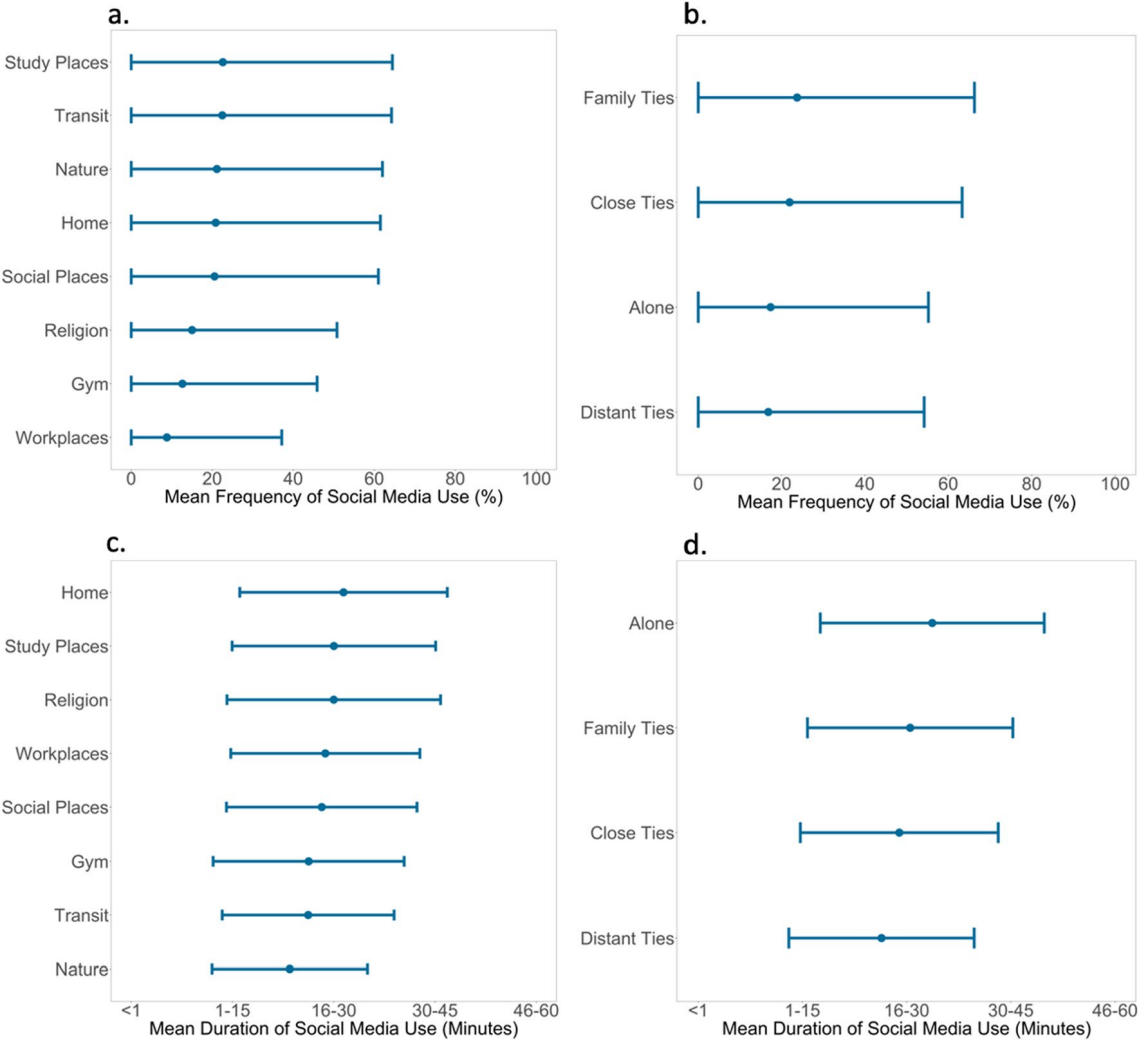
Social media usage across physical and social contexts. *Note*: Social media use frequency represents the % of observations in which people reported using social media. Duration represents the average time spent in the past hour using social media platforms. Points depict the mean level of social media use in different contexts. Error bars depict one standard deviation above and below the mean for social media use in different contexts.

People used social media platforms most frequently around family members and close ties (Fig. 3a), and while in study places and in transit (Fig. 3b). They were used least frequently when people were alone and around distant ties, and while in the gym and workplace. In terms of the degree of use, people used social media platforms for longer durations than their own average when they were alone and around family ties (Fig. 3c), and while they were at home and in study places (Fig. 3d). Similarly, people used social media for shorter durations than their own average when they were around close ties and distant ties, and while in transit and in nature.

### Physical context moderators

#### Loneliness

**Figure 4:**
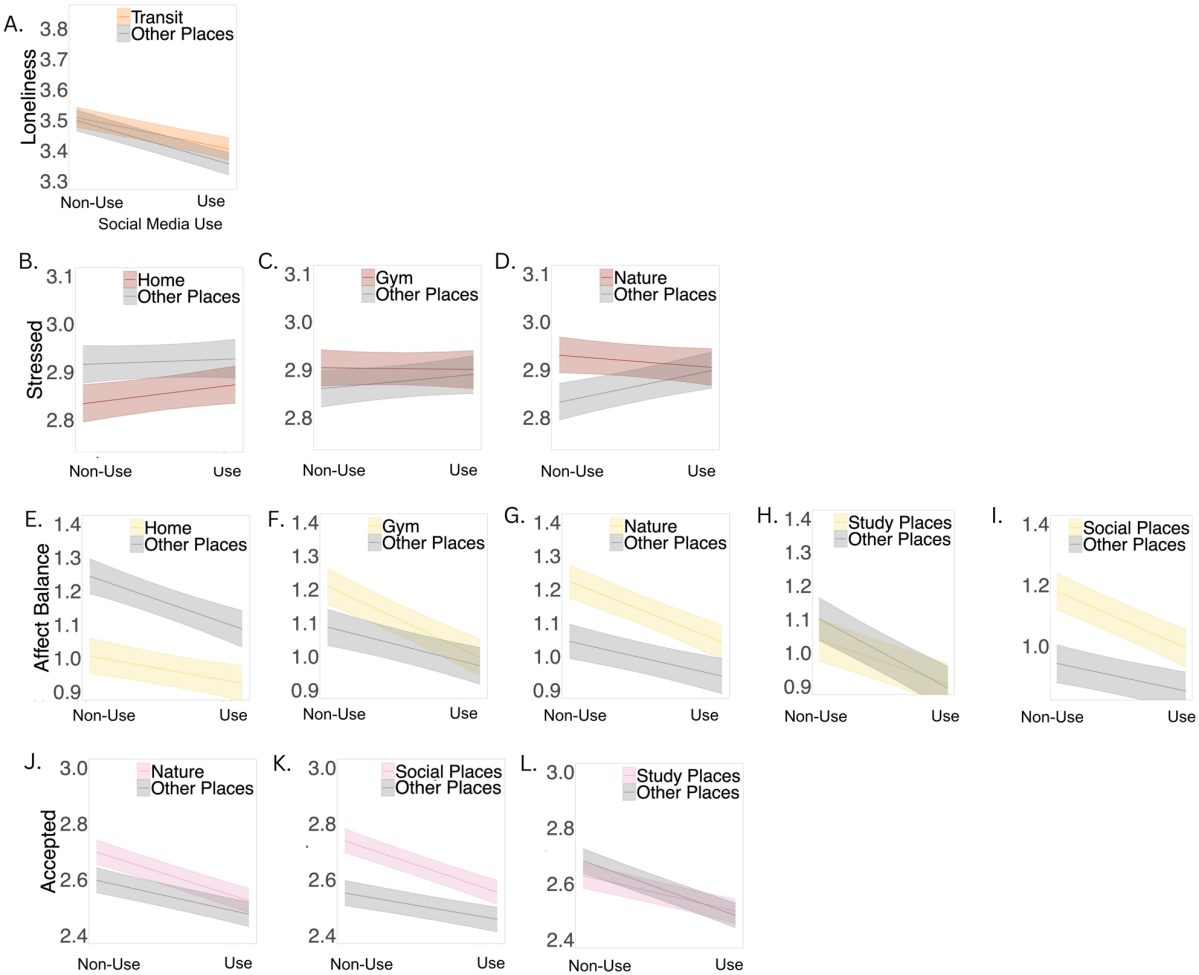
Physical context moderators of social media use vs non-use). *Note*: Stress and loneliness were reverse coded such that higher values indicate lower levels of stress and loneliness. Bands depict 95% confidence interval of simple slope estimates.

**Figure 5:**
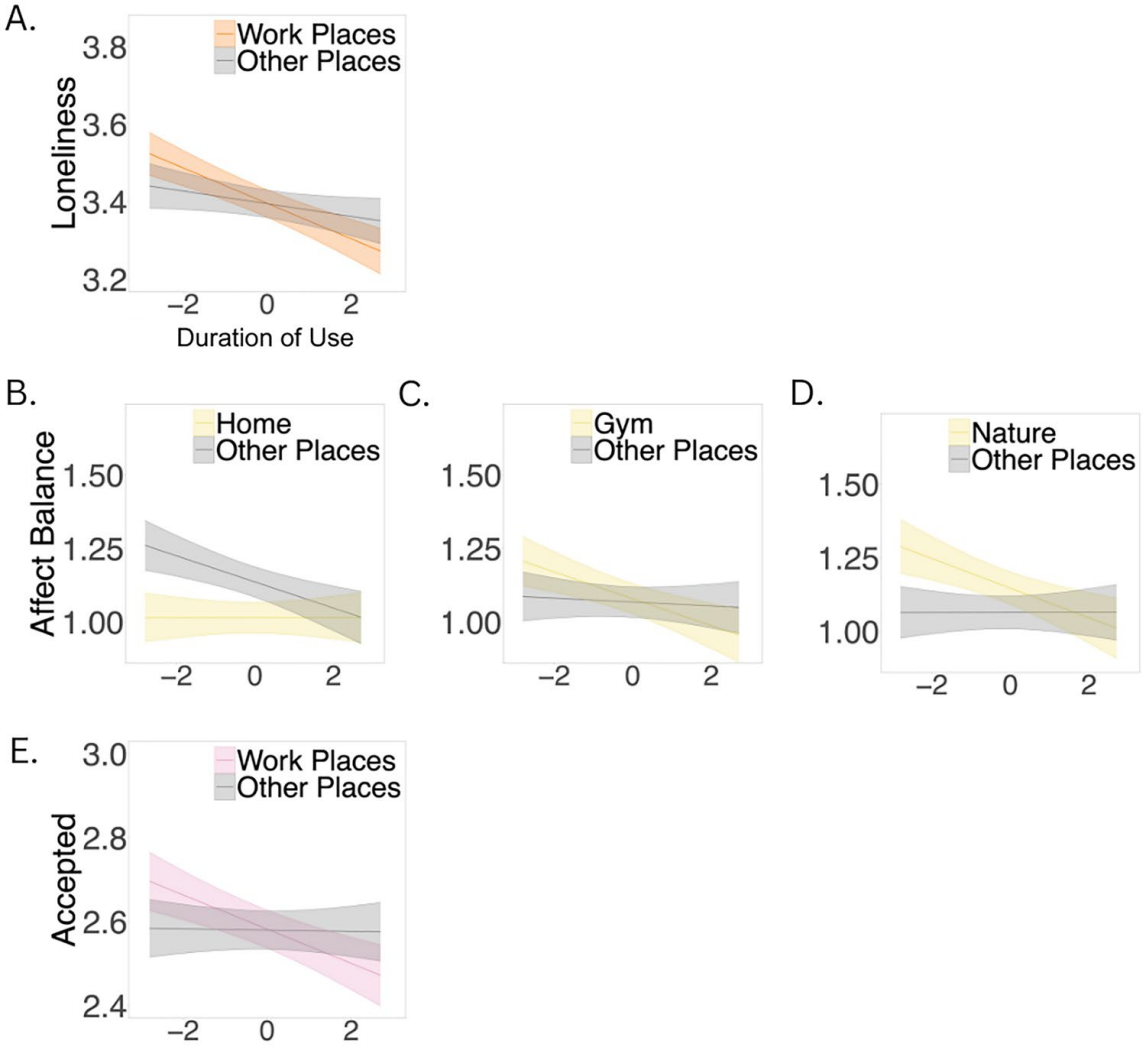
Physical context moderators of duration of use. *Note*: Stress and loneliness were reverse coded such that higher values indicate lower levels of stress and loneliness. Bands depict 95% confidence interval of simple slope estimates. Values less than 0 denote below person-specific average duration of social media use. Values greater than 0 denote above person-specific average duration of social media use.

People reported feeling lonelier after using social media while they were in transit (Table S37, Fig. 4a), as compared to using social media in other places. Similarly, people reported feeling lonelier after using social media for longer durations of time than their own average in work-places (Table S37, Fig. 5a) as compared to other places.

#### Stress

People reported feeling greater stress after using social media when they were in nature (Table S38, Fig. 4d), as compared to using social media in other places.

#### Affect balance

People reported a greater negative affect balance after using social media when they were outside the home (Table S39, Fig. 4e), as compared to using social media when they were at home. Specifically, people reported a greater negative affect balance after using social media when they were at the gym (Table S39, Fig. 4f), in nature (Table S39, Fig. 4g) and in social places (Table S39, Fig. 4h), as compared to using social media in other places. Conversely, people reported a positive affect balance after using social media when they were in study places (Table S39, Fig. 4i), as compared to using social media in other types of contexts. In terms of the degree of use, people reported a greater negative affect balance after using social media for longer durations than their own average outside their home (Table S39, Fig. 5b) as compared to using social media for longer durations than their own average while they were at home. Similarly, people reported a greater negative affect balance after using social media for longer durations than their own average when they were at the gym (Table S39, Fig. 5c) and in nature (Table S39, Fig. 5d), as compared to using social media for longer durations than their own average in other places.

#### Feelings of being accepted

People reported lower feelings of being accepted after using social media in nature (Table S40, Fig. 4j), social places (Table S40, Fig. 4k) and study places (Table S40, Fig. 4l), as compared to using social media in other places. In terms of the degree of use, people reported lower feelings of being accepted after using social media for longer durations than their own average when they were at their workplaces (Table S40, Fig. 5e), as compared to after using social media for longer durations than their own average in other places.

### Social context moderators

#### Stress

**Figure 6:**
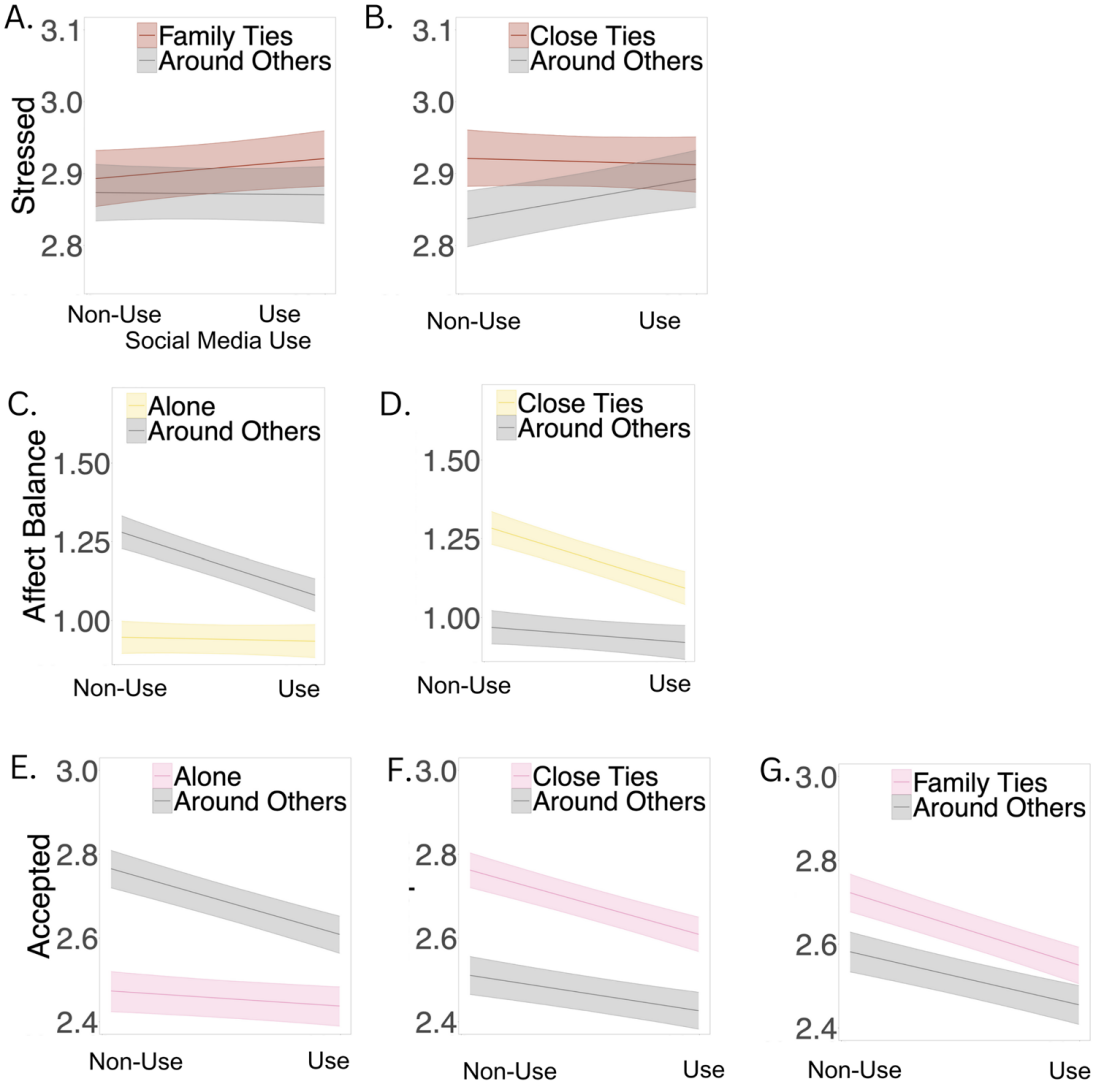
Social context moderators of social media use vs non-use. *Note*: Stress and loneliness were reverse coded such that higher values indicate lower levels of stress and loneliness. Bands depict 95% confidence interval of simple slope estimates.

**Figure 7:**
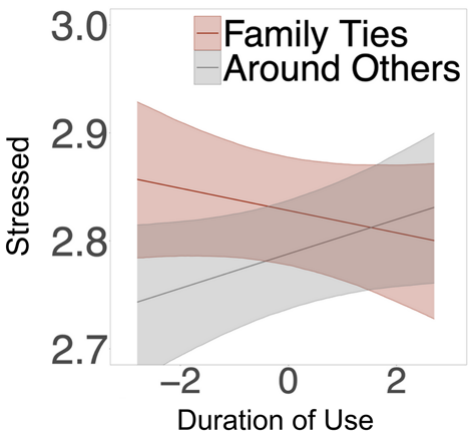
Social context moderators  duration of use. *Note*: Stress and loneliness were reverse coded such that higher values indicate lower levels of stress and loneliness. Bands depict 95% confidence interval of simple slope estimates.

People reported lower feelings of stress when using social media around people that were not close ties (Table S38, Fig. 6b), as compared to using social media around people who were close ties. Similarly, people reported feeling greater stress after using social media for longer durations than their own average when they were around family ties (Table S38, Fig. 7), as compared to after using social media around other people.

#### Affect balance

People reported a greater negative affect balance after using social media around others (Table S39, Fig. 6c), as compared to using social media alone. Specifically, people reported a greater negative affect balance after using social media around close ties (Table S39, Fig. 6d), as compared to after using social media around other people.

#### Feelings of being accepted

People reported lower feelings of being accepted after using social media around others (Table S40, Fig. 6e), as compared to using social media alone. Specifically, people reported lower feelings of being accepted after using social media around family ties (Table S40, Fig. 6f) and close ties (Table S40, Fig. 6g), as compared to after using social media around other people.

## Discussion

Our primary goal in this research paper was to introduce and empirically investigate the construct of social media sensitivity. On average, social media use was associated with worse wellbeing, but this average association masked a great deal of heterogeneity in whether and the degree to which social media was associated with lower wellbeing within and across individuals. To examine how social media sensitivities varied across and within people, we analyzed a large experience sampling dataset collected from young adults over the course of a month using a mixture of frequentist and Bayesian multilevel modelling techniques. We found support for the idea that (1) people experience different social media sensitivities that are associated with their psychological dispositions, and (2) the physical and social contexts in which people use social media platforms are associated with their social media sensitivity in the moment. Our analytic approach allowed us to comprehensively disaggregate between and within-person effects, and account for many different control variables (including preceding wellbeing states) to precisely estimate effect sizes for people’s social media sensitivity.

We have three sets of findings that are consequential for research on the psychological effects of social media use and the regulation of social media platforms. First, we found evidence that supports the idea that there is generally a negative, albeit a small association between social media use and social and affective wellbeing. That is, on average, people reported feeling greater negative affect, lower feelings of being accepted, greater stress, and greater feelings of loneliness after using social media, compared to after not using social media. A similar pattern of results was observed for the duration of social media use. When people used social media for longer durations than usual (in reference to their own average duration as a baseline), they reported lower social (e.g., feelings of being accepted, loneliness) and affective wellbeing (e.g., affect balance, stress), compared to when they used social media for shorter durations than usual. However, we also observed considerable heterogeneity between people in the relationship between social media use and wellbeing outcomes. Hence, we corroborate many recent findings that indicate that social media and wellbeing associations tend to differ from person to person [e.g.,^6,34^].

Much of the past research has studied social media’s effects on affective wellbeing in adolescents. We build upon this past work in two concrete ways. First, we investigated associations between social media and wellbeing in a large sample of young adults and found similar levels of heterogeneity as those observed in adolescents. Second, we investigated the heterogeneity in associations between social media use an social wellbeing, which is particularly relevant for platforms meant to facilitate the formation and maintenance of social relationships^35^. We found qualitatively similarly levels of heterogeneity in associations between social media use and social and affective wellbeing, suggesting that heterogenous associations between social media use and wellbeing are not limited to affective operationalizations of the latter construct. Hence, this set of findings addresses concerns raised by past scholars how about different operationalizations of wellbeing might influence findings about associations between social media and wellbeing^36,37^.

Our results revealed that people with dispositional psychological vulnerabilities (e.g., higher depression, lower satisfaction with life) experienced greater negative social media sensitivities across the social and affective wellbeing outcomes, in comparison to people who were less psychologically vulnerable. These findings corroborate recent research that has similarly found psychologically vulnerable people to asymmetrically suffer from poor mental health as a result of using social media^38,39^. We replicated this pattern of results using experience sampling methods. In contrast, most past research on this topic that has focused on panel data [e.g.,^3,40^]. Hence the convergent findings are particularly noteworthy given that they imply, for example, that vulnerabilities might magnify the negative effects of social media use on wellbeing while simultaneously buffering any positive effects^40–42^.

Being in specific physical contexts (e.g., in social places and in nature) while using social media was also associated with greater negative social media sensitivity on average. Similarly, being in the company of certain people (e.g., close ties, family ties) was associated with greater negative social media sensitivity on average. In contrast, people were least sensitive to social media when they used social media platforms at home or while alone. These findings suggest that not all social media use results in negative wellbeing outcomes. By focusing on understanding the context in which social media use is occurring, researchers and policymakers can gain a better understanding of *when, where* and *around whom* social media use is detrimental, and when it might be beneficial. Furthermore, the construct of social media sensitivity allows researchers to accommodate differences in the relationship between social media use and wellbeing across three dimensions: from person-to-person, from context-to-context, and from time-to-time. In the current paper, we focused on understanding how the relationship between social media and wellbeing varies from person-to-person and context-to-context. Future research should examine how the relationship varies from time-to-time.

We further highlight two important observations. First, we failed to find specific dispositions that make people positively sensitive to social media use. That is, there were no dispositional traits (e.g., extraversion) that made people more likely to feel better after using social media platforms. Similarly, there were no physical contexts that made people more likely to feel better after using social media. These patterns of findings were also true for social context: there were no people around whom social media use was associated with positive wellbeing outcomes. A second limitation was that the negative social media sensitivity that we did observe were small in terms of effect sizes (in the range of 0.02–0.08). It is possible that these small associations, and the absence of positive social media sensitivity findings are being driven by (a) idiosyncrasies of our data samples (collected primarily during the pandemic) and (b) by analytical decisions made about comparison groups (e.g., being in social places vs. other places as compared to being in social places vs. at home). An analysis of individual participants’ data might reveal that certain people have positive social media sensitivities in certain contexts, however at the average level, these associations are masked. In any case, it is not particularly surprising that the observed effect sizes were small, given that affective and social wellbeing are psychological outcomes that are being independently affected by many different processes^43^. Indeed, our findings corroborate a large body of research that finds small to near-zero associations between social media use and wellbeing, especially as examined in the temporal domain of everyday life^44–46^. Hence, the possibility remains that the true effect size of interest is truly in the range of small effect sizes—a possibility that is difficult to ignore given that our work has greater between-person power as compared to previous experience sampling research on social media use and wellbeing.

For public policy legislation, it is essential to consider how legislation can be targeted to prioritize the wellbeing of the most vulnerable. Since associations between social media and wellbeing were heterogenous across people and operationalizations of wellbeing, any universally applicable legislation (e.g., wherein social media use is discouraged) will be more effective for some people (e.g., those who have a negative social media sensitivity). Hence, the conversation surrounding the legislation of social media should focus on determining which segments of the population would benefit from external regulation of social media platforms (e.g., those with psychologically vulnerable dispositions) instead of focusing overtly on the unrealistic end-goal of benefiting all segments of the population by implementing blanket policies. Similarly, public policy initiatives can focus on informational campaigns that educate the public about social media’s heterogenous effects, while social media companies can increase transparency regarding which of their users may be sensitive to positive or negative effects of their platforms and provide tools to assist with self-regulation of social media use^47,48^.

The main weakness of the current research is that we operationalized social media use via a binary (use vs non-use) and duration-based measure using self-report methods. This is a limitation for several reasons. First, self-report measures of social media use have been shown to correlate only weakly with objective measures of social media use obtained from log data [e.g.,^49,50^]. This weakness is caveated with newer literature that suggests that self-report measures of social media use have comparable predictive validity for psychological outcomes as compared to digital trace data. Hence, we expect that that many of our findings are likely to replicate with more objective measures of social media use^51^. Moreover, in relying on self-report measures, we capture people’s social media mindsets which have shown proximate associations with wellbeing outcomes^52^. Similarly, self-report measures allow us to collect social media data from iPhone users, which is typically impossible to do in an objective manner without ethical concerns [e.g., this requires “jailbreaking” out of the default system settings:,^53^]. Lastly, by relying on self-report methods, we were able to capture social media use across desktop and smartphones, which is typically difficult to do with more objective traces of data.

Second, global self-report measures of social media use prevent an adequate understanding of platform-level differences. By using global self-report measures, we failed to capture heterogeneity in wellbeing associations that was explained by what people were doing on specific social media platforms. This can be a fertile ground for future research. Specifically, future research can capture on-platform behavior and content consumption on social media platforms using novel screenshot techniques [e.g.,^54^]. Such work could extend the contributions of the current research to create a portrait of how people’s dispositions and context of social media use relate to what they are doing on social media platforms. Similarly, to better understand the mechanisms that explain our observed patterns of findings, researchers can conduct additional experience sampling studies that capture the extent to which social media use *interfered with* or was *conducive to* the contexts that participants occupied. For instance, it is possible that using social media around others results in lowered wellbeing as a result of an active interference process^55^, where in people’s attention is divided between their devices and their social company. These variables can be explicitly measured by asking participants to report the perceived utility of their social media use.

Our research is also limited in that it samples participants from highly industrialized Western settings. Hence, we emphasize that our findings generalize to college-going young adults in the United States but may not generalize to other cross-sections of the American population or populations outside of the United States. Indeed, since social media platforms are used ubiquitously across the globe, which has led to recent calls for research examining their effects on wellbeing in diverse populations in the Global South^56^. Since we are interested in examining the moderating role of physical and social context on social media and wellbeing associations, we cannot ignore the role played by cultural differences in shaping people’s patterns of social media use, their global feelings of wellness, as well as the plurality of physical and social contexts they inhabit. Even though we collected data from highly industrialized Westernized settings, our sample composition is quite ethnically diverse: over 50% of our sample is composed of people of color (see Methods sections). Future research could further alleviate these weaknesses by collecting data from representative panels of non-Western social media users to validate the construct of social media sensitivity in understudied populations.

These weaknesses aside, our paper makes a pivotal contribution to social media and wellbeing research. We formulate the construct of social media sensitivity to examine three dimensions along which social media and wellbeing associations differ: *who* is using these platforms, *where* these platforms are being used, and *around whom* these platforms are being used. Our results suggest that each of these three dimensions introduce heterogeneity in social media sensitivity. Specifically, we find that the associations between social media use and wellbeing differ significantly across people and the contexts in which they use social media. Psychologically vulnerable people are more likely to suffer from the negative repercussions of using social media. Social media use is not detrimental to wellbeing when used alone and at home but is associated with negative changes in wellbeing when used in social and natural places. As a result of our contribution, future research is better prepared to understand the myriad factors that contribute to social media sensitivity, especially using combinations of novel methodologies (e.g., objective trace data) and causal designs (e.g., field-experiments).

## Materials and methods

### Participants

Participants were college students recruited from an introductory psychology class at University of Texas at Austin. Research protocols were approved by the university IRB (Protocol No. 2018-07-0035). The datasets analyzed in this paper were collected in Fall 2020 (exploratory sample: n = 920, observations = 73,284) and Spring 2021 (confirmatory sample: n = 764, observations = 55,903). Prior to data analysis, we followed an initial data procedure to ensure that only high-quality experience sampling occasions were retained in the final sample:

1. ESM surveys completed too quickly. We computed a threshold based on the number of questions completed in each ESM survey (by multiplying this number by 0.5 s). We subsequently filtered any reports that were completed faster than the threshold.
2. Participant-specific ESM surveys completed too close in time to each other (less than 60 min after the previous report).
3. ESM surveys that took too long to complete (more than 60 min).
4. Participants who indicated in the post-survey that they had not been truthful in the ESM surveys.

We then excluded participants who failed to complete more than 65% of the total required experience sampling reports to gain credit for the assignment. As a final step, we removed participants who were older than 24 years of age since our target population of interest was young adults.

Initial data cleaning procedures resulted in the removal of 32 participants corresponding to 5245 observations for the exploratory sample. Subsequently, we removed 18 participants corresponding to 1528 observations who were older than 24 years of age from the exploratory sample. Finally, we removed 49 participants corresponding to 577 observations because they failed to complete more than 65% of the total number of experience sampling questions required to gain credit for the assignment. Hence, the final sample size of the exploratory sample was 821 participants corresponding to 65,934 observations.

Similarly, initial data cleaning procedures led to the removal of 20 participants corresponding to 3343 observations from the confirmatory sample. We additionally removed 10 participants corresponding to 837 observations who were older than 24 years of age from the confirmatory sample. Finally, we removed 53 participants corresponding to 837 observations because they failed to complete more than 65% of the total number of experience sampling questions required to gain credit for the assignment. Hence, the final size of the confirmatory sample was 681 participants corresponding to 51,500 observations.

In the combined sample consisting of both the exploratory and confirmatory sample, most participants identified as female (69.1%) with a mean age of 18.7 years and were enrolled in either their first (58.7%) or second year of college (26.1%). Most participants identified as Anglo/White (32.5%), Asian/Asian American (21.7%), Hispanic/Latino (25.6%) and African Americans (5.1%).

### Procedure

Participants completed a demographic survey during the first week of the semester and a range of personality questionnaires during other weeks of the semester. Participants received seven daily ESM surveys for up to four weeks. Participants received full credit if they provided at-least fourteen days of data with at least four surveys on each of those days. Participants downloaded an application onto their smartphones which sent periodic push notifications to participants about pending surveys. The notifications were programmed to arrive at semi-random times within seven 120-min blocks between 8 am and 10 pm, with a minimum time window of 60 min between each consecutive notification. Participants were permitted to complete surveys on their phones or computers, but notifications expired by the end of each block. Hence, the average time window between two consecutive surveys within the same day was 163 min. The surveys were distributed in the following pattern throughout the day: 22% during the morning, 24% during the midday, 27% during midday, 27% during afternoon and 24% during the evening. All participating students were compensated with class credit and personalized feedback reports that summarized their social media use trends and psychological wellbeing patterns over the course of the semester.

### Momentary measures

#### Wellbeing

Wellbeing was measured using seven adjectives: “happy”, “sad”, “valued and accepted by others”, “lonely”, “worried”, “angry” and “stressed”. Participants responded to each scale using a 1–4 Likert scale (ranging from “not at all” to “a great deal”). The question stem asked participants to indicate their feelings “right now”, explicitly capturing momentary wellbeing at the time of the ESM. The adjectives “angry”, “worried”, “happy”, and “sad” were borrowed directly from past work^31^. Following past research^31,57^, we computed momentary affect balance by subtracting the “happy” score from the arithmetic mean score of “sad”, “worried” and “angry”. We treated momentary affect balance and stress as indicators of affective wellbeing. Conversely, we treated momentary feelings of being accepted and loneliness as indicators of social wellbeing. We reverse scored stress and loneliness variables such that higher values on different wellbeing outcomes all indicated “positive wellbeing”. Hence, higher values of loneliness, stress, affect balance and feelings of being accepted all indicate *positive wellbeing.*

#### Social media use (vs non-use)

During each ESM survey, participants indicated the activities they had engaged in the past hour using a “select all that apply” multiple choice question. The question stem was “During the PAST HOUR, I spent time doing the following activities (check all that apply)”. The response options consisted of 19 different behaviors (summarized in Table S41) of which one was “Using social media”. We created a new categorical dummy variable that indexed all instances of social media use (vs non-use). All instances of social media use were labelled as “1”. All instances of non-social media use were labelled as “0”. All missing values were preserved. We also assessed participants’ engagement in multitasking as the *number of activities* performed in the past hour, specifically calculated as the number of activities they indicated performing during the past hour.

#### Duration of use

If participants selected “using social media” as an activity, branch logic displayed a follow-up question asking participants to rate the duration of their social media use in the past hour on a 4-point scale: 1 = 1–15 min, 2 = 16–30 min, 3 = 31–45 min, 4 = 46–60 min.

#### Context

At each ESM survey, participants indicated, via “select all that apply” multiple choice response, who they were with during the last hour (social context) and what places they had been in during the last hour (physical context). The social context question stem was: “During the PAST HOUR, I spent time with the following people in-person (check all that apply)”. Participants could indicate having spent time with 8 different categories of people (see Table S41). Based on past research, we created a set of 4 dummy variables from the 8 response options: alone, with family ties, with close ties, and with distant ties (see Table S41). Dummy variables were created such that target social context categories were encoded with a 1, and non-target categories were encoded with a 0 (e.g., Alone = 1, With Other People = 0). All missing values were preserved.

Similarly, the physical context question stem was: “During the PAST HOUR, I spent time in the following places (check all that apply)”. People could indicate having spent time in 13 different types of places. Motivated by theoretical frameworks about psychologically salient physical and social context^55,58,59^, we created a set of 8 dummy variables from these responses: home, social places, natural places, work places, transit, study places, religious places (see Table S41). Dummy variables were created such that target places were encoded with a 1, while non-target places were encoded with a 0 (e.g., Home = 1, Other Places = 0). All missing values were preserved.

### Individual differences and dispositional measures

#### Demographics

Participants’ *age* and *sex* was measured in the demographics survey administered prior to the experience sampling component of the study.

#### Personality traits

Participants’ Big Five Personality Traits were measured before the start of the experience sampling component of the study. We used the BFI-2 instrument, which consists of 60-items answered using a 5-point Likert Scale^28,60^. The Big Five Traits measure uses the average of 12 items to measure interindividual differences in extraversion, agreeableness, neuroticism, conscientiousness, and openness. Extraversion captures differences in individuals’ tendency to be gregarious, assertive, energetic, and talkative. Agreeableness captures differences in individuals’ tendency to be trustful, altruistic, modest, and warm. Neuroticism capture one’s tendency to be anxious, angry/hostile, depressed, self-conscious, and impulsive. Conscientiousness captures one’s tendency to be competent, orderly, dutiful achievement striving, self-discipline and deliberative. Finally, Openness captures one’s tendency to be imaginative, have an aesthetic proclivity, preference for variety and curiosity.

#### Dispositional wellbeing

The following wellbeing tendencies were measured before the start of the experience sampling component of the study: depressive symptoms, satisfaction with life, loneliness, and affect balance.

Depressive symptoms were measured using the Center for Epidemiological Studies-Depression scale that asks participants to indicate a variety of depressive symptoms in the preceding week, including loneliness, poor appetite, and restless sleep^30^. Higher values corresponded with greater depression symptomatology.

Satisfaction with life was measured using the Diener Satisfaction with Life Scale, as the average of responses provided on a 1 (strongly disagree) to 7 (strongly agree) scale to 5 statements that operationalizes a holistic perspective towards their lived and ideal lives^32^. People’s satisfaction with life scores are calculated by taking an arithmetic mean of the 5 items of the scale. Higher values corresponded with greater satisfaction with life.

Loneliness was measured using the UCLA loneliness scale, that measures participants’ agreement with 9 statements that ask about the frequency with which participants experience moments of social connection or social disconnection^61^. Participants responded using a 1 (*I never feel this way)* to 4 (*I always feel this way)* scale. Upon reverse scoring a subset of the statements, the final score is calculated by computing an arithmetic mean of all response items. Higher values corresponded to greater loneliness.

Dispositional affect balance was measured using a modified form of the SOEP scales (e.g., Angry, Worried, Happy, Sad, Enthusiastic, Relaxed:^31^). People indicated the extent to which they felt angry, worried, happy, sad, enthusiastic, and relaxed using a 1 (*Very rarely)—*5 (*Very often)* scale. People’s dispositional affect balance was computed by subtracting the mean of their negative emotion scores (e.g., angry, worried, sad, relaxed) from their positive emotion scores (e.g., happy, sad, enthusiastic and relaxed). Hence, positive values corresponded to greater positive affect whereas negative values corresponded to greater negative affect.

### Modelling strategy

Data analyses for each of the three research questions was done using multilevel models that accommodated the nested nature of the data (repeated measures nested within-persons). Following usual practice within and between-person effects are disentangled through person-mean centering of all time-varying (Level 1) predictor variables and sample-mean centering of all person-level (Level 2) predictor variables^62^. Much of the past research has disproportionately focused on examining between-person associations between social media and wellbeing. Hence, to build upon past research, we were especially interested in cross-level interactions (e.g., the extent to which between-person differences in psychological dispositions explain within-person relationships between social media and wellbeing) and within-person moderation effects (e.g. comparing people’s feelings of wellbeing after using social media as compared to when they did not use social media).

For main effect analysis, our general analytic approach consisted of specifying separate models wherein: (1) one of our four possible wellbeing outcomes is specified as a dependent variable and (2) one of two possible operationalizations of social media use are included as predictors. As a result, a total of 8 main effects models were computed. Similarly, for moderation analysis, our general analytic approach consisted of specifying separate models wherein: (1) one of four possible wellbeing outcomes (e.g., feelings of being accepted, loneliness, stress and affect balance) is specified as a dependent variable, (2) one of two possible operationalizations of social media use (e.g., use vs non-use; duration of use) are included as predictors and (3) one of nine possible dispositional variables or one of eleven possible context variables are included as moderators. As a result, a total of 168 moderator models were computed. Across both main effects, dispositional moderators and contextual moderators, a total of 172 models were computed.

### What is the relationship between social media use and wellbeing in young adults’ daily lives?

We used frequentist linear regression models in lme4^63^ with random intercepts and random slopes allowed to vary across participants to determine the extent to which social media use and wellbeing are related at the within and between-person levels:

$$\begin{aligned} Wellbeing _{ti} & = \beta_{0i} + \beta_{1i} SocialMedia _{ti} + \beta_{2i} Wellbeing _{{\left( {t - 1} \right)i}} + \beta_{3i} DurationSinceLastResponse _{ti} \\ & \quad + \beta_{4i} Wellbeing _{{\left( {t - 1} \right)i}} DurationSinceLastResponse _{ti} + \beta_{5i} NumberofActivities _{ti} \\ & \quad + \beta_{6i} Weekend _{ti} + \beta_{7i} StudyDay _{ti} + \beta_{8i} Sample _{ti} + e_{ti} \\ \end{aligned}$$

where wellbeing at occasion *t* for person *i* is modeled as a function of a person-specific intercept coefficient ${\beta }_{0i}$ that indicates the individual’s prototypical level of wellbeing, a set of person-specific coefficients ${\beta }_{1i}$ to ${\beta }_{7i}$ that indicate the within-person associations between the predictor variables and wellbeing, and residual ${e}_{ti}$ that are assumed normally distributed with standard deviation ${\sigma }_{e}.$ The person-specific coefficients are then modeled as a function of between-person differences. Specifically,

$${\beta }_{0i}={\gamma }_{00}+{\gamma }_{01}{\text{ SocialMedia }}_{i}+{\gamma }_{02}{\text{ Age }}_{i}+{\gamma }_{03}{\text{ Sex }}_{i}+{\gamma }_{04}{\text{ NumberOfResponses }}_{i} + {u}_{0i}$$

$${\beta }_{1i}={\gamma }_{10}+{u}_{1i}$$

$${\beta }_{2i}={\gamma }_{20}$$

$${\beta }_{3i}={\gamma }_{30}$$

$${\beta }_{4i}={\gamma }_{40}$$

$${\beta }_{5i}={\gamma }_{50}$$

$${\beta }_{6i}={\gamma }_{60}$$

$${\beta }_{7i}={\gamma }_{70}$$

where the gammas are sample-level parameters that indicate the intercept and effects for the prototypical individuals, and the residuals ${u}_{0i}$ and ${u}_{1i}$ are residual individual differences in intercept and the within-person association between social media use and wellbeing that are assumed multivariate normal with standard deviations ${\sigma }_{u0}$ and ${\sigma }_{u1}$ and correlation ${r}_{{\sigma }_{u0}{\sigma }_{u1}}$. Of specific interest are the ${\gamma }_{10}$ and ${\sigma }_{u1}$ parameters. We define social media sensitivity as referring to the ${\gamma }_{10}$ parameter whereas ${\sigma }_{u1}$ captures the person-level heterogeneity of social media sensitivity.

### What is the relationship between dispositional traits and social media sensitivity?

Dispositional moderators were fit using the lme4 package^63^ in R with restricted maximum likelihood and missing data (< 0.1%) was treated as missing at random. Statistical significance was evaluated at alpha = 0.05. Dispositional moderators were modelling using the following model:

$${Wellbeing}_{ti}={\beta }_{0i}+{\beta }_{1i}S{ocialMedia}_{ti}+{e}_{ti}$$

$${\beta }_{0i}={\gamma }_{00}+{\gamma }_{01}S{ocialMedia}_{i}+ {\gamma }_{02}{Personality}_{i}+ {\gamma }_{03}S{ocialMedia}_{i}{Personality}_{i}+{u}_{0i}$$

$${\beta }_{1i}={\gamma }_{10}+{\gamma }_{11}{Personality}_{i} +{u}_{1i}$$

where ${\gamma }_{03}$ is the between-person interaction and ${\gamma }_{11}$ is the cross-level interaction.

### What is the relationship between context of use and social media sensitivity?

Random intercepts and slopes were specified for social media, context, and their resulting interactions, resulting in complex models that did not converge in a frequentist framework. Hence, we used a Bayesian paradigm for model estimation to facilitate model convergence. The move to Bayesian estimation allowed us to examine the extent to which multiple momentary contexts moderate the relationship between momentary social media use and wellbeing. The expanded model is specified by the following equation:

$$\begin{aligned} Wellbeing_{ti} & = \beta_{0i} + \beta_{1} SocialMedia_{ti} + \beta_{2} SocialMedia_{i} \\ & \quad + \beta_{3} AffectiveWellbeing_{{\left( {t - 1} \right)i}} + \beta_{4} DurationSinceLastResponse_{ti} \\ & \quad + \beta_{5} AffectiveWellbeing_{{\left( {t - 1} \right)i}} DurationSinceLastResponse_{ti} \\ & \quad + \beta_{6} NumberofActivities_{ti} + \beta_{7} Weekend_{ti} + \beta_{8} StudyDay_{ti} \\ & \quad + \beta_{9} SocialMedia_{ti} Context_{ti} + \beta_{10} Sample_{ti} + e_{ti} \\ \end{aligned}$$

$${\beta }_{0i}={\gamma }_{00}+{\gamma }_{01}S{ocialMedia}_{i}+ {\gamma }_{02}{Context}_{i}+ {\gamma }_{03}S{ocialMedia}_{i}{Context}_{i}+{\gamma }_{04}{Age}_{i}+{\gamma }_{06}{Sex}_{i}+ {\gamma }_{07}{NumberOfResponses}_{i}+{u}_{0i}$$

$${\beta }_{1i}={\gamma }_{10}+{\gamma }_{11}{Context}_{i} +{u}_{1i}$$

where ${\beta }_{9}$ is the within-person interaction, ${\gamma }_{03}$ is the between-person interaction and ${\gamma }_{11}$ is the cross-level interaction between average social media use and context. Contextual moderators were fit using the brms package^64^ with 12,000 iterations (half warm-up) since convergence was not possible with lme4. All models were specified with normal priors and achieved convergence with 12,000 iterations. We did not specify hyperpriors to constrain the hyperparameters of the model. We used the Monte Carlo Markov Chain sampler when fitting the Bayesian models. Specifically, we used 4 separate chains in each model and ensured that they each yielded stable estimates by examining the R-hat values (all values did not exceed 1.01)^65^. We determined which models yielded coefficients that were larger than 0 by examining the corresponding 95% credibility intervals for each estimate.

### Supplementary Information

Supplementary Information.
