# Supplementary material for: Variation in social media sensitivity across people and contexts
Source: Sci Rep. 2024 Mar 19;14:6571. doi: 10.1038/s41598-024-55064-y (PMC10951328; doi:10.1038/s41598-024-55064-y)
Supplement: Supplementary file 1 — Supplementary Information. [file 41598_2024_55064_MOESM1_ESM.pdf]

Supplemental Materials

Social Media Sensitivity

Table of Contents

1. Exploratory Findings: Dataset 1 from Fall 2020
    a. Main Effects of Social Media Use on Wellbeing
    b. Cross-Level Findings for Psychological Dispositions
    c. Between-Person Findings for Psychological Dispositions (*denoted by BP)
    d. Within-Person Findings for Physical and Social Context (*denoted by WP)
    e. Cross-Level Findings for Physical and Social Context
    f. Between-Person Findings for Physical and Social Context (*denoted by BP)

2. Confirmatory Findings: Dataset 2 from Spring 2021
    a. Main Effects of Social Media Use on Wellbeing
    b. Cross-Level Findings for Psychological Dispositions
    c. Between-Person Findings for Psychological Dispositions (*denoted by BP)
    d. Within-Person Findings for Physical and Social Context (*denoted by WP)
    e. Cross-Level Findings for Physical and Social Context
    f. Between-Person Findings for Physical and Social Context (*denoted by BP)

3. Mega-analytic Findings: Pooled Datasets from Fall 2020 & Spring 2021
    a. Between-Person Findings for Psychological Dispositions (*denoted by BP)
    b. Cross-level Findings for Physical and Social Context
    c. Between-Person Findings for Physical and Social Context (*denoted by BP)

4. Comparisons between Exploratory and Confirmatory Samples

5. Measured Variables

1. Dataset 1 (Fall 2020): Exploratory Findings

1a. Main Effects of Social Media Use on Wellbeing

Figure S1: Social Media Sensitivity Across Social and Affective Wellbeing (Exploratory Sample)

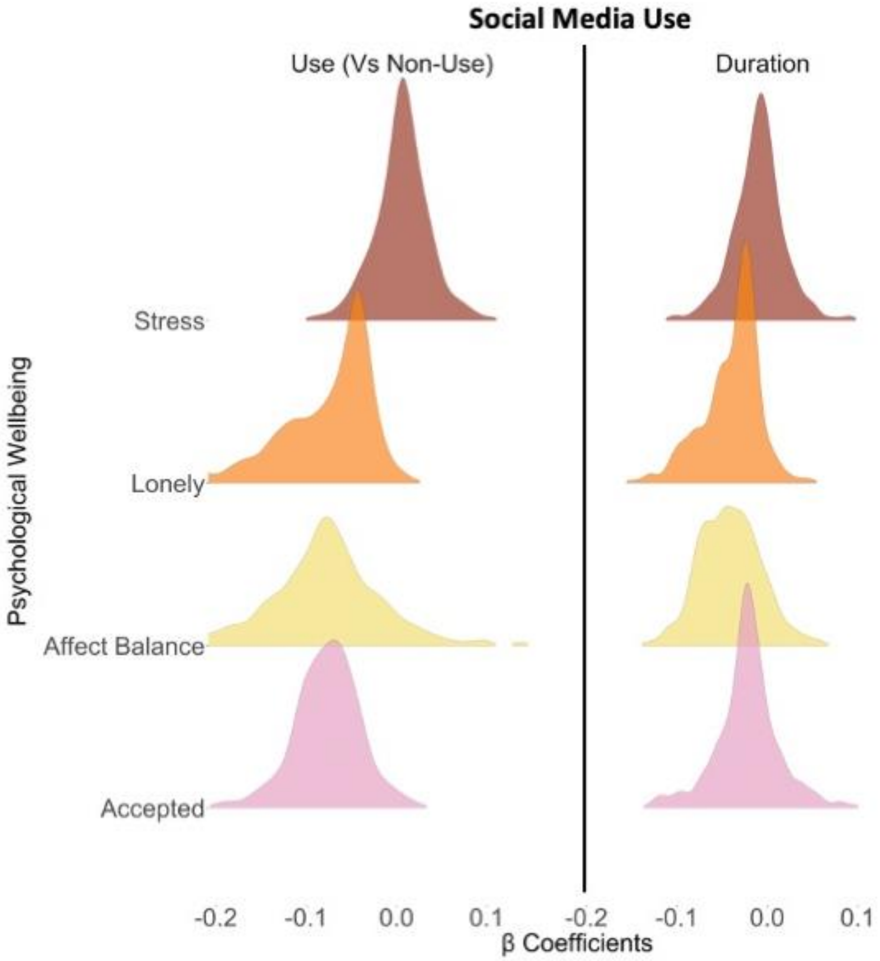

1b. Cross-Level Findings for Psychological Dispositions

Loneliness. People who were higher in depression, dispositional loneliness, and have a dispositional negative affect reported feeling lonelier after using social media as compared to their counterparts.
Stress. People who were higher in dispositional loneliness reported feeling more stressed after using social media platforms as compared to people who were lower in dispositional loneliness.
Affect Balance. People who were higher in depression reported a negative affect balance after using social media platforms as compared to people who were lower in depression. People who were higher in depression reported a negative affect balance after using social media for longer durations than their own average, as compared to people who were lower in depression.

1c. Between-Person Findings for Psychological Dispositions

Loneliness. People who were higher in dispositional loneliness and loneliness and tended to use social media for long durations of time reported greater feelings of loneliness generally, as compared to people who were lower in dispositional loneliness and depression, and tended to use social media for long durations of time.
Stress. People who were higher in dispositional loneliness and tended to use social media frequently reported greater feelings of stress generally, as compared to people who were lower in dispositional loneliness and tended to use social media frequently. People who were higher in depression and tended to use social media for longer durations of time reported greater feelings of stress generally, as compared to people who were lower in depression.
Affect Balance. People who were higher in depression and tended to use social media frequently and for long durations of time reported a negative affect balance generally, as compared to people who were lower in depression and tended to use social media frequently for long durations of time.
Feelings of Being Accepted. People who were higher in conscientiousness and tended to use social media for long durations of time reported lower feelings of being accepted generally, as compared to people who were lower in conscientiousness and tended to use social media for long durations of time.

1d. Within-Person Findings for Physical and Social Context

Loneliness. People reported greater feelings of loneliness after using social media around close ties, as compared to using social media around other types of interaction partners.
Stress. People reported greater feelings of after using social media for longer durations of time as compared to their own average when they were in transit, as compared to using social media for long durations in other types of places.
Affect Balance. People reported a lower affect balance after using social media in social places, as compared to using social media in other types of locations. Similarly, people reported a lower affect balance after using social media when they were with others, specifically with close ties and family ties, as compared to using social media when they were alone or with distant ties.
Feelings of Being Accepted. People reported lower feelings of being accepted after using social media for longer durations than their own average in social places, as compared to other types of locations. People also reported lower feelings of being accepted after using

social media for longer durations than their average when they were in work places, as compared to after using social media for longer durations than their average in other types of places.

1e. Cross-level Findings for Physical and Social Context

Loneliness. People who were around distant ties frequently reported greater feelings of loneliness after using social media as compared to people who were around distant ties less frequently. People who tended to be in nature more often reported greater feelings of loneliness after using social media more, and for longer durations of time as compared to their own average, relative to people who were in nature less often in general. People who were in transit more often reported greater feelings of loneliness after using social media as compared to people who were in transit less generally.
Stress. People who were around distant ties frequently reported greater feelings of stress after using social media for longer durations of time as compared to their own average, relative to people who were around distant ties less frequently.
Feelings of Being Accepted. People who were in nature frequently reported feeling less accepted after using social media as compared to people who were in nature less frequently. Similarly, people who were around others more often reported lower feelings of being accepted after using social media for longer durations as compared to people who were alone more often.

1f. Between-Person Findings for Physical and Social Context

Stress. People who were in work contexts frequently and reported using social media for long durations of time reported generally greater stress, as compared to people who were in work contexts frequently and reported using social media for short durations of time.
Affect Balance. People who were in transit frequently and reported using social media for long durations of time reported a generally negative affect balance, as compared to people who were in transit frequently and reported using social media for short durations of time.
Feelings of Being Accepted. People who were in transit contexts frequently and used social media frequently reported feeling more accepted generally as compared to people who were in transit contexts frequently and reported using social media frequently. Similarly, people who were around family ties more often and reported using social media frequently time felt more accepted generally, as compared to people who were around family ties less often and reported using social media frequently.

Table S1: Dispositional Wellbeing Moderators (Momentary Loneliness) (Exploratory Sample)

	**Affect Balance**	**Depression**	**Loneliness**	**Satisfaction with Life**
**Intercept**	3.456***	3.48***	3.456***	3.455***
	3.374***	3.391***	3.37***	3.374***
**Dispositional Trait**	0.147***	-0.042***	-0.383***	0.027***
	0.152***	-0.108***	-0.388***	0.028***
**Dispositional Trait (BP) x Social Media Use (BP)**	-0.066	0.004	0.126	-0.012
	0.017	-0.048**	-0.143*	0.007
**Dispositional Trait (BP) x Social Media Use (WP)**	0.014**	-0.004**	-0.029*	0.002^{T}
	-0.002	-0.003	0.013	-0.001

Note: White rows correspond to the effects of social media use (vs non-use) and grey rows correspond to the effects of social media use duration. ***p<0.001, **p<0.01, *p<0.05,^{T} p<0.1

Table S2: Big Five Personality Trait Moderators (Momentary Loneliness) (Exploratory Sample)

	**Openness**	**Conscientiousness**	**Extraversion**	**Agreeableness**	**Neuroticism**
**Intercept**	3.466***	3.464***	3.468***	3.466***	3.47***
	3.392***	3.395***	3.394***	3.391***	3.399***
**Dispositional Trait**	-0.016	0.045	0.065*	0.074^{T}	-0.234
	0.006	0.033***	0.082*	0.091*	-0.26***
**Dispositional Trait (BP) x Social Media Use (BP)**	0.021	-0.109	0.132	0.03	-0.301^{T}
	-0.027	0.047	0.04	-0.006	-0.076^{T}
**Dispositional Trait (BP) x Social Media Use (WP)**	0.014	0.003	0.018^{T}	0.011	-0.024*
	-0.001	0.003	-0.014	-0.023	0.006

Note: White rows correspond to the effects of social media use (vs non-use) and grey rows correspond to the effects of social media use duration. ***p<0.001, **p<0.01, *p<0.05,^{T} p<0.1

Table S3: Dispositional Moderators (Momentary Stress) (Exploratory Sample)

	Affect Balance	Depression	Loneliness	Satisfaction with Life
**Intercept**	2.789***	2.792***	2.789***	2.787***
	2.795***	2.801***	2.794***	2.797***
**Dispositional Trait**	0.171***	-0.04***	-0.304***	0.031***
	0.163***	-0.042***	-0.251***	0.028***
**Dispositional Trait (BP) x Social Media Use (BP)**	-0.114	0.0000	0.584*	-0.034
	0.006	-0.013*	-0.114	0.009
**Dispositional Trait (BP) x Social Media Use (WP)**	-0.004	-0.001	0.045**	-0.001
	0.000	0.000	0.003	-0.002

Note: White rows correspond to the effects of social media use (vs non-use) and grey rows correspond to the effects of social media use duration. ***p<0.001, **p<0.01, *p<0.05,[T] p<0.1

Table S4: Big Five Personality Traits Moderators (Momentary Stress) (Exploratory Sample)

	Openness	Conscientiousness	Extraversion	Agreeableness	Neuroticism
**Intercept**	2.789***	2.789***	2.791***	2.789***	2.788***
	2.8***	2.802***	2.806***	2.804***	2.808***
**Dispositional Trait**	-0.042	0.036***	0.061***	0.067***	-0.294***
	-0.07	0.015***	0.054	0.047	-0.295***
**Dispositional Trait (BP) x Social Media Use (BP)**	-0.175	0.211	0.119	-0.418^{T}	-0.002
	-0.012	0.039	0.07	0.097	-0.071
**Dispositional Trait (BP) x Social Media Use (WP)**	-0.01	-0.023	-0.021	-0.002	-0.008
	-0.01	0.018	-0.002	-0.01	-0.005

Note: White rows correspond to the effects of social media use (vs non-use) and grey rows correspond to the effects of social media use duration. ***p<0.001, **p<0.01, *p<0.05,^{T} p<0.1

Table S5: Dispositional Wellbeing Moderators (Momentary Affect Balance) (Exploratory Sample)

	Affect Balance	Depression	Loneliness	Satisfaction with Life
**Intercept**	0.969***	0.98***	0.978***	0.976***
	1.016***	0.976***	0.96***	0.971***
**Dispositional Trait**	0.348***	-0.078***	-0.772***	0.07***
	0.335***	-0.081***	-0.733***	0.071***
**Dispositional Trait (BP) x Social Media Use (BP)**	-0.004	0.043^{T}	0.206	-0.034
	-0.014	-0.015*	-0.044	0.006
**Dispositional Trait (BP) x Social Media Use (WP)**	0.003	-0.005**	0.004	0.001
	0.001	0.000	-0.003	-0.002

Note: White rows correspond to the effects of social media use (vs non-use) and grey rows correspond to the effects of social media use duration. ***p<0.001, **p<0.01, *p<0.05,^{T} p<0.1

Table S6: Big Five Personality Trait Moderators (Momentary Affect Balance) (Exploratory Sample)

	Openness	Conscientiousness	Extraversion	Agreeableness	Neuroticism
**Intercept**	0.971***	0.966***	0.971***	0.972***	0.969***
	0.976***	0.977***	0.976***	0.98***	0.982***
**Dispositional Trait**	0.041	0.222***	0.385***	0.306***	-0.524***
	0.063	0.2***	0.384***	0.255***	-0.53***
**Dispositional Trait (BP) x Social Media Use (WP)**	0.294	0.022	0.249	0.164*	-0.261
	0.012	0.066	0.011	0.092	-0.085
**Dispositional Trait (BP) x Social Media Use (BP)**	0.008	-0.02	0	-0.036	-0.018
	0.006	0.012	0.004	-0.03	-0.009

Note: White rows correspond to the effects of social media use (vs non-use) and grey rows correspond to the effects of social media use duration. ***p<0.001, **p<0.01, *p<0.05,^{T} p<0.1

Table S7: Dispositional Wellbeing Moderators (Momentary Feelings of Being Accepted) (Exploratory Sample)

	Affect Balance	Depression	Loneliness	Satisfaction with Life
**Intercept**	2.535***	2.531***	2.533***	2.533***
	2.508***	2.513***	2.5***	2.51***
**Dispositional Trait**	0.217***	-0.055***	0.05***	0.05***
	0.216***	-0.053***	-0.66***	0.049***
**Dispositional Trait (BP) x Social Media Use (BP)**	-0.041	0.055*	-0.035	-0.035
	0.017	-0.019***	-0.089	0.011*
**Dispositional Trait (BP) x Social Media Use (WP)**	0.002	-0.001	0	0
	0.007	-0.002	-0.003	-0.001

Note: White rows correspond to the effects of social media use (vs non-use) and grey rows correspond to the effects of social media use duration. ***p<0.001, **p<0.01, *p<0.05,^{T} p<0.1

Table S8: Big Five Personality Trait Moderators (Momentary Feelings of Being Accepted) (Exploratory Sample)

	**Openness**	**Conscientiousness**	**Extraversion**	**Agreeableness**	**Neuroticism**
**Intercept**	2.526***	2.524***	2.525***	2.531***	2.523***
	2.501***	2.51***	2.498***	2.504***	2.504***
**Dispositional Trait**	0.128**	0.219***	0.389***	0.312***	-0.347***
	0.163***	0.174***	0.373***	0.291***	-0.346***
**Dispositional Trait (BP) x Social Media Use (BP)**	0.183	-0.199	0.137	0.403	-0.104
	-0.045	0.17**	0	0.069	-0.07
**Dispositional Trait (BP) x Social Media Use (WP)**	0.008	-0.018	0.007	-0.013	-0.005
	-0.017	-0.004	-0.004	-0.022	-0.008

Note: White rows correspond to the effects of social media use (vs non-use) and grey rows correspond to the effects of social media use duration. ***p<0.001, **p<0.01, *p<0.05

Table S9: Physical Context Moderators(Momentary Loneliness) (Exploratory Sample)

	**Home**	**Nature**	**Gym**	**Religion**	**Social**	**Work**	**Transit**	**Study**
**Intercept**	2.54 [2.484,2.595]	2.549 [2.495,2.604]	2.544 [2.487,2.599]	2.546 [2.49,2.603]	2.543 [2.492,2.596]	2.546 [2.492,2.599]	2.544 [2.488,2.597]	2.543 [2.489,2.599]
	2.524 [2.465,2.584]	2.528 [2.464,2.592]	2.518 [2.456,2.581]	2.517 [2.456,2.579]	2.518 [2.458,2.578]	2.518 [2.457,2.58]	2.535 [2.473,2.597]	2.522 [2.461,2.583]
**Context (WP)**	-0.207 [-0.233,-0.182]	0.195 [0.159,0.231]	0.081 [0.03,0.132]	0.146 [-0.017,0.314]	0.281 [0.25,0.312]	0.03 [-0.03,0.091]	0.14 [0.11,0.17]	-0.035 [-0.073,0.004]
	-0.166 [-0.223,-0.109]	0.128 [0.049,0.207]	0.083 [-0.071,0.239]	-0.001 [-0.484,0.469]	0.217 [0.157,0.277]	0.024 [-0.113,0.165]	0.166 [0.108,0.225]	-0.056 [-0.132,0.021]
**Context (BP)**	-0.255 [-0.455,-0.058]	2.244 [1.133,3.359]	1.77 [0.193,3.37]	6.381 [0.509,12.383]	0.718 [0.321,1.115]	0.377 [-0.43,1.19]	1.23 [0.418,2.048]	0.16 [-0.078,0.393]
	-0.372 [-0.581,-0.162]	-0.021 [-0.142,0.101]	1.595 [-0.07,3.26]	3.668 [-2.036,9.372]	0.799 [0.39,1.215]	0.41 [-0.386,1.213]	1.272 [0.439,2.124]	0.182 [-0.055,0.426]
**Social Media Use (vs Non-Use) (WP) x Context (WP)**	0.031 [-0.024,0.087]	-0.05 [-0.129,0.029]	-0.059 [-0.215,0.095]	-0.17 [-0.556,0.218]	-0.093 [-0.161,-0.024]	0.071 [-0.066,0.206]	-0.014 [-0.083,0.054]	0.041 [-0.029,0.111]
	0.034 [-0.026,0.095]	-0.033 [-0.139,0.076]	-0.024 [-0.212,0.163]	0.39 [-0.395,1.107]	-0.03 [-0.104,0.044]	-0.184 [-0.362,-0.008]	-0.014 [-0.089,0.062]	-0.038 [-0.119,0.042]
**Social Media Use (vs Non-Use) (WP) x Context (BP)**	-0.008 [-0.069,0.053]	-0.338 [-0.622,-0.054]	-0.023 [-0.551,0.501]	-1.203 [-2.951,0.543]	-0.089 [-0.219,0.04]	0.008 [-0.26,0.273]	-0.193 [-0.42,0.036]	0.034 [-0.034,0.102]
	-0.032 [-0.097,0.032]	-0.351 [-0.666,-0.03]	0.166 [-0.464,0.786]	-0.961 [-3.125,1.208]	-0.123 [-0.25,0.006]	-0.219 [-0.527,0.078]	-0.37 [-0.6,-0.144]	0.062 [-0.01,0.134]
**Social Media Use (vs Non-Use) (BP) x Context (BP)**	-0.176 [-1.378,1.05]	-2.933 [-6.989,1.238]	0.483 [-10.322,11.468]	-15.433 [-41.681,11.435]	1.224 [-1.318,3.737]	1.091 [-4.374,6.676]	-0.235 [-3.131,2.651]	0.081 [-1.191,1.359]
	-0.12 [-0.423,0.184]	1.266 [-0.626,3.129]	1.303 [-0.558,3.175]	4.465 [-4.906,13.806]	-0.456 [-1.137,0.231]	0.427 [-0.791,1.651]	1.589 [0.33,2.834]	0.288 [-0.053,0.629]

Table S10: Social Context Moderators (Momentary Loneliness) (Exploratory Sample)

	**Alone**	**Family Ties**	**Close Ties**	**Distant Ties**
Intercept	2.525 [2.469,2.579]	2.543 [2.487,0.182]	2.536 [2.48,2.589]	2.542 [2.486,2.598]
	2.512 [2.453,2.571]	2.519 [2.459,2.581]	2.512 [2.452,2.571]	2.514 [2.451,2.577]
Context (WP)	-0.268 [-0.291,-0.246]	0.192 [0.164,0.22]	0.304 [0.275,0.332]	0.027 [0.002,0.051]
	-0.204 [-0.241,-0.166]	0.136 [0.091,0.182]	0.23 [0.185,0.275]	0.032 [-0.021,0.083]
Context (BP)	-0.926 [-1.138,-0.71]	-0.027 [-0.278,0.233]	0.586 [0.386,0.778]	0.379 [-0.13,0.877]
	-0.891 [-1.125,-0.666]	-0.028 [-0.285,0.231]	0.645 [0.439,0.852]	0.312 [-0.224,0.851]
Social Media Use (vs Non-Use) (WP) x Context (WP)	0.088 [0.054,0.122]	-0.053 [-0.097,-0.01]	-0.069 [-0.108,-0.029]	-0.003 [-0.06,0.056]
	0.014 [-0.027,0.054]	0.036 [-0.01,0.082]	-0.029 [-0.079,0.021]	-0.023 [-0.09,0.044]
Social Media Use (vs Non-Use) (WP) x Context (BP)	0.08 [-0.19,0.068]	-0.013 [-0.084,0.057]	-0.039 [-0.097,0.021]	-0.21 [-0.37,-0.049]
	0.044 [-0.024,0.113]	-0.016 [-0.09,0.059]	-0.025 [-0.087,0.036]	-0.11 [-0.292,0.069]
Social Media Use (vs Non-Use) (BP) x Context (BP)	-0.562 [-1.654,0.514]	-0.64 [-1.884,0.592]	0.483 [-0.547,1.511]	1.749 [-1.129,4.543]
	-0.086 [-0.406,0.239]	0.63 [0.271,0.996]	-0.099 [-0.394,0.184]	-0.171 [-0.952,0.628]

Table S11: Physical Context Moderators (Momentary Stress) (Exploratory Sample)

	**Home**	**Nature**	**Gym**	**Religion**	**Social**	**Work**	**Transit**	**Study**
**Intercept**	2.789<br>[2.739,2.836]	2.794<br>[2.745,2.841]	2.792<br>[2.744,2.839]	2.789<br>[2.743,2.835]	2.792<br>[2.744,2.838]	2.791<br>[2.742,2.84]	2.792<br>[2.745,2.841]	2.79<br>2.742,2.838]
	2.799<br>[2.744,2.855]	2.796<br>[2.739,2.854]	2.797<br>[2.74,2.854]	2.795<br>[2.739,2.851]	2.793<br>[2.737,2.849]	2.798<br>[2.743,2.853]	2.787<br>[2.732,2.843]	2.797<br>[2.741,2.853]
**Context (WP)**	-0.095<br>[-0.114,-0.075]	0.11<br>[0.079,0.141]	0.139<br>[0.09,0.189]	0.107<br>[-0.019,0.233]	0.107<br>[0.084,0.131]	0.031<br>[-0.014,0.075]	0.084<br>[0.059,0.109]	-0.04<br>[-0.069,-0.011]
	-0.056<br>[-0.108,-0.004]	0.002<br>[-0.079,0.082]	0.18<br>[-0.012,0.376]	-0.111<br>[-0.474,0.254]	0.067<br>[0.015,0.118]	0.124<br>[-0.002,0.252]	0.08<br>[0.018,0.142]	-0.035<br>[-0.1,0.029]
**Context (BP)**	0.033<br>[-0.141,0.211]	1.624<br>[0.693,2.552]	2.05<br>[0.68,3.406]	-0.687<br>[-5.96,4.531]	-0.069<br>[-0.42,0.272]	-0.122<br>[-0.808,0.577]	0.681<br>[-0.038,1.409]	-0.095<br>[-0.303,0.113]
	0.044<br>[-0.147,0.236]	1.126<br>[-0.057,2.289]	1.523<br>[0.059,2.962]	-0.57<br>[-5.52,4.416]	-0.145<br>[-0.523,0.234]	-0.237<br>[-0.944,0.454]	0.148<br>[-0.611,0.91]	-0.078<br>[-0.289,0.139]
**Social Media Use (vs Non-Use) (WP) × Context (WP)**	0.009<br>[-0.042,0.061]	-0.075<br>[-0.154,0.003]	0.005<br>[-0.173,0.181]	-0.132<br>[-0.488,0.219]	-0.035<br>[-0.094,0.023]	0.003<br>[-0.001,0.004]	0.036<br>[-0.037,0.107]	0.063<br>[-0.011,0.135]
	0.035<br>[-0.026,0.095]	-0.017<br>[-0.121,0.084]	0.001<br>[-0.231,0.245]	-0.125<br>[-0.619,0.399]	-0.042<br>[-0.106,0.021]	-0.034<br>[-0.226,0.15]	-0.084<br>[-0.159,-0.011]	-0.019<br>[-0.099,0.062]
**Social Media Use (vs Non-Use) (WP) × Context (BP)**	-0.01<br>[-0.07,0.051]	-0.002<br>[-0.293,0.29]	0.043<br>[-0.493,0.577]	0.305<br>[-1.397,2.02]	-0.019<br>[-0.146,0.109]	0<br>[-0.391,0.13]	-0.184<br>[-0.409,0.048]	0.021<br>[-0.044,0.086]
	0.013<br>[-0.049,0.075]	0.049<br>[-0.266,0.36]	0.423<br>[-0.202,1.049]	0.403<br>[-1.448,2.252]	0.042<br>[-0.083,0.17]	-0.064<br>[-0.361,0.235]	-0.08<br>[-0.302,0.143]	-0.032<br>[-0.1,0.036]
**Social Media Use (vs Non-Use) (BP) × Context (BP)**	0.008<br>[-0.106,0.121]	-1.916<br>[-5.552,1.878]	7.253<br>[-2.053,16.397]	2.847<br>[-20.373,25.796]	-1.072<br>[-3.389,1.264]	0.138<br>[-5.401,4.566]	-0.584<br>[-3.11,2.017]	0.095<br>[-1.013,1.217]
	0.056<br>[-0.026,0.137]	0.079<br>[-1.556,1.767]	0.27<br>[-1.379,1.934]	2.573<br>[-5.459,10.51]	-0.54<br>[-1.148,0.074]	-1.12<br>[-2.212,-0.039]	-1.366<br>[-2.493,-0.235]	0.084<br>[-0.223,0.386]

Note: White rows correspond to the effects of social media use (vs non-use) and grey rows correspond to the effects of social media use duration. 95% Bayesian credible intervals are presented below each co-efficient in square brackets.

Table S12: Social Context Moderators (Momentary Stress) (Exploratory Sample)

	Alone	Family Ties	Close Ties	Distant Ties
**Intercept**	2.787 [2.739,2.835]	2.79 [2.742,2.837]	2.792 [0.058,0.259]	2.789 [2.741,2.838]
	2.794 [2.737,2.85]	2.793 [2.737,2.849]	2.793 [0.017,0.194]	2.792 [2.738,2.847]
**Context (WP)**	-0.049 [-0.063,-0.035]	0.017 [-0.002,0.036]	0.075 [0.058,0.092]	0.001 [-0.022,0.024]
	-0.01 [-0.027,0.007]	0.018 [-0.019,0.056]	0.054 [0.017,0.09]	-0.024 [-0.079,0.031]
**Context (BP)**	-0.062 [-0.262,0.136]	-0.087 [-0.309,0.14]	0.087 [-0.088,0.259]	-0.221 [-0.688,0.236]
	-0.034 [-0.245,0.186]	0.027 [-0.202,0.251]	0.004 [-0.188,0.194]	-0.399 [-0.871,0.079]
**Social Media Use (vs Non-Use) (WP) x Context (WP)**	0.007 [-0.029,0.043]	0.012 [-0.032,0.057]	-0.008 [-0.048,0.033]	-0.041 [-0.1,0.019]
	0.011 [-0.028,0.051]	-0.006 [-0.059,0.047]	-0.015 [-0.058,0.028]	-0.013 [-0.085,0.06]
**Social Media Use (vs Non-Use) (WP) x Context (BP)**	0.031 [-0.038,0.101]	0.036 [-0.036,0.108]	-0.047 [-0.107,0.013]	-0.172 [-0.337,-0.01]
	-0.027 [-0.096,0.041]	-0.002 [-0.075,0.072]	0.028 [-0.033,0.089]	-0.004 [-0.176,0.169]
**Social Media Use (vs Non-Use) (BP) x Context (BP)**	-0.198 [-1.239,0.827]	0.528 [-0.52,1.569]	-0.32 [-1.228,0.593]	1.246 [-1.185,3.776]
	0.074 [-0.22,0.364]	-0.059 [-0.386,0.277]	-0.053 [-0.318,0.213]	-0.471 [-1.158,0.226]

Note: White rows correspond to the effects of social media use (vs non-use) and grey rows correspond to the effects of social media use duration. 95% Bayesian credible intervals are presented below each co-efficient in square brackets.

Table S13: Physical Context Moderators (Momentary Affect Balance) (Exploratory Sample)

	Home	Nature	Gym	Religion	Social	Work	Transit	Study
**Intercept**	0.981 [0.918,1.043]	0.987 [0.924,1.048]	0.987 [0.926,1.049]	0.985 [0.923,1.048]	0.986 [0.92,1.052]	0.984 [0.923,1.047]	0.99 [0.927,1.053]	0.986 [0.923,1.048]
	1.036 [0.981,1.092]	1.035 [0.977,1.094]	1.022 [0.966,1.078]	1.021 [0.966,1.078]	1.022 [0.966,1.078]	1.021 [0.965,1.076]	1.025 [0.968,1.083]	1.023 [0.969,1.078]
**Context (WP)**	-0.28 [-0.312,-0.248]	0.311 [0.264,0.358]	0.335 [0.257,0.414]	0.139 [-0.05,0.325]	0.365 [0.326,0.404]	-0.016 [-0.087,0.056]	0.187 [0.147,0.227]	-0.019 [-0.07,0.032]
	-0.191 [-0.241,-0.142]	0.194 [0.113,0.276]	0.033 [-0.111,0.178]	-0.078 [-0.39,0.232]	0.266 [0.214,0.318]	-0.08 [-0.211,0.056]	0.139 [0.08,0.199]	0.002 [-0.06,0.066]
**Context (BP)**	-0.212 [-0.444,0.013]	2.539 [1.328,3.754]	3.119 [1.315,4.933]	8.984 [2.377,15.551]	0.623 [0.172,1.07]	0.542 [-0.354,1.433]	2.076 [1.156,3.001]	-0.028 [-0.299,0.24]
	-0.259 [-0.449,-0.07]	2.526 [1.587,3.472]	2.414 [1.054,3.782]	3.343 [-0.861,7.484]	0.536 [0.155,0.922]	0.235 [-0.483,0.957]	1.15 [0.476,1.833]	0.031 [-0.182,0.248]
**Social Media Use (vs Non-Use) (WP) × Context (WP)**	0.049 [-0.022,0.119]	-0.058 [-0.169,0.055]	-0.166 [-0.392,0.06]	-0.244 [-0.756,0.271]	-0.109 [-0.196,-0.022]	0.057 [-0.13,0.245]	-0.078 [-0.177,0.019]	0.094 [-0.008,0.194]
	0.07 [0.015,0.124]	-0.123 [-0.226,-0.019]	-0.195 [-0.38,-0.012]	-0.276 [-0.758,0.2]	-0.062 [-0.13,0.005]	0 [-0.002,0.002]	-0.05 [-0.125,0.024]	-0.019 [-0.097,0.059]
**Social Media Use (vs Non-Use) (WP) × Context (BP)**	0.024 [-0.066,0.113]	-0.262 [-0.693,0.164]	0.241 [-0.537,1.005]	0.514 [-2.004,3.03]	-0.086 [-0.28,0.107]	-0.131 [-0.516,0.254]	-0.135 [-0.477,0.209]	0.046 [-0.055,0.147]
	0.013 [-0.054,0.079]	-0.13 [-0.423,0.163]	-0.262 [-0.811,0.301]	-0.458 [-2.19,1.271]	-0.049 [-0.18,0.083]	-0.002 [-0.003,-0.001]	-0.077 [-0.283,0.123]	-0.039 [-0.114,0.036]
**Social Media Use (vs Non-Use) (BP) × Context (BP)**	-0.209 [-0.391,-0.025]	-3.578 [-8.31,1.23]	7.001 [-4.829,18.959]	-17.993 [-48.18,12.585]	-0.845 [-3.892,2.202]	0.266 [-6.208,6.647]	-2.293 [-5.775,1.123]	0.122 [-1.337,1.6]
	-0.007 [-0.086,0.072]	1.336 [-0.216,0.005]	-0.154 [-1.846,1.545]	1.215 [-4.37,6.827]	-0.72 [-1.298,-0.155]	-0.094 [-0.255,0.064]	0.095 [-0.965,1.178]	0.035 [-0.262,0.33]

Note: White rows correspond to the effects of social media use (vs non-use) and grey rows correspond to the effects of social media use duration. 95% Bayesian credible intervals are presented below each co-efficient in square brackets.

Table S14: Social Context Moderators (Momentary Affect Balance) (Exploratory Sample)

	Alone	Family Ties	Close Ties	Distant Ties
**Intercept**	0.967 [0.904,1.029]	0.981 [0.918,1.045]	0.977 [0.317,0.886]	0.979 [0.916,1.045]
	1.012 [0.957,1.067]	0.098 [0.062,0.135]	0.209 [0.168,0.251]	0.044 [-0.017,0.104]
**Context (WP)**	-0.277 [-0.301,-0.253]	0.15 [0.118,0.181]	0.349 [0.317,0.381]	0.073 [0.04,0.107]
	-0.191 [-0.241,-0.142]	0.194 [0.113,0.276]	0.033 [-0.111,0.178]	-0.078 [-0.39,0.232]
**Context (BP)**	-0.951 [-1.198,-0.707]	-0.007 [-0.304,0.291]	0.663 [0.43,0.886]	0.214 [-0.359,0.786]
	-0.828 [-1.042,-0.612]	0.039 [-0.189,0.267]	0.64 [0.451,0.832]	0.461 [-0.004,0.923]
**Social Media Use (vs Non-Use) (WP) × Context (WP)**	0.115 [0.065,0.164]	-0.06 [-0.123,0.002]	-0.076 [-0.135,-0.017]	-0.08 [-0.161,0.001]
	0.023 [-0.016,0.063]	0.005 [-0.04,0.05]	-0.015 [-0.062,0.032]	-0.046 [-0.116,0.026]
**Social Media Use (vs Non-Use) (WP) × Context (BP)**	0.057 [-0.044,0.156]	0.049 [-0.059,0.157]	-0.054 [-0.142,0.035]	-0.112 [-0.355,0.133]
	0.047 [-0.027,0.121]	0.022 [-0.055,0.099]	-0.058 [-0.122,0.007]	-0.038 [-0.202,0.126]
**Social Media Use (vs Non-Use) (BP) × Context (BP)**	-0.216 [-1.531,1.09]	0.075 [-1.33,1.461]	0.018 [-1.144,1.196]	1.517 [-1.876,4.763]
	0.07 [-0.215,0.358]	0.025 [-0.299,0.347]	0.041 [-0.235,0.322]	-0.53 [-1.222,0.142]

Note: White rows correspond to the effects of social media use (vs non-use) and grey rows correspond to the effects of social media use duration. 95% Bayesian credible intervals are presented below each co-efficient in square brackets.

Table S15: Physical Context Moderators (Momentary Feelings of Being Accepted) (Exploratory Sample)

	Home	Nature	Gym	Religion	Social	Work	Transit
Intercept	2.54 [2.484,2.595]	2.549 [2.495,2.604]	2.544 [2.487,2.599]	2.546 [2.49,2.603]	2.543 [2.492,2.596]	2.546 [2.492,2.599]	2.544 [2.488,2.597]
	2.524 [2.465,2.584]	2.528 [2.464,2.592]	2.518 [2.456,2.581]	2.517 [2.456,2.579]	2.518 [2.458,2.578]	2.518 [2.457,2.58]	2.535 [2.473,2.597]
Context (WP)	-0.207 [-0.233,-0.182]	0.195 [0.159,0.231]	0.081 [0.03,0.132]	0.146 [-0.017,0.314]	0.281 [0.25,0.312]	0.03 [-0.03,0.091]	0.14 [0.11,0.17]
	-0.166 [-0.223,-0.109]	0.128 [0.049,0.207]	0.083 [-0.071,0.239]	-0.001 [-0.484,0.469]	0.217 [0.157,0.277]	0.024 [-0.113,0.165]	0.166 [0.108,0.225]
Context (BP)	-0.255 [-0.455,-0.058]	2.244 [1.133,3.359]	1.77 [0.193,3.37]	6.381 [0.509,12.383]	0.718 [0.321,1.115]	0.377 [-0.43,1.19]	1.23 [0.418,2.048]
	-0.372 [-0.581,-0.162]	2.049 [0.76,3.369]	1.595 [-0.07,3.26]	3.668 [-2.036,9.372]	0.799 [0.39,1.215]	0.41 [-0.386,1.213]	1.272 [0.439,2.124]
Social Media Use (vs Non-Use) (WP) × Context (WP)	0.031 [-0.024,0.087]	-0.05 [-0.129,0.029]	-0.059 [-0.215,0.095]	-0.17 [-0.556,0.218]	-0.093 [-0.161,-0.024]	0.071 [-0.066,0.206]	-0.014 [-0.083,0.054]
	0.034 [-0.026,0.095]	-0.033 [-0.139,0.076]	-0.024 [-0.212,0.163]	0.39 [-0.395,1.107]	-0.03 [-0.104,0.044]	-0.184 [-0.362,-0.008]	-0.014 [-0.089,0.062]
Social Media Use (vs Non-Use) (WP) × Context (BP)	-0.008 [-0.069,0.053]	-0.338 [-0.622,-0.054]	-0.023 [-0.551,0.501]	-1.203 [-2.951,0.543]	-0.089 [-0.219,0.04]	0.008 [-0.26,0.273]	-0.193 [-0.42,0.036]
	-0.032 [-0.097,0.032]	-0.351 [-0.666,-0.03]	0.166 [-0.464,0.786]	-0.961 [-3.125,1.208]	-0.123 [-0.25,0.006]	-0.219 [-0.527,0.078]	-0.37 [-0.6,-0.144]
Social Media Use (vs Non-Use) (BP) × Context (BP)	-0.126 [-0.273,0.021]	-2.933 [-0.136,0.091]	0.483 [-10.322,11.468]	-15.433 [-41.681,11.435]	1.224 [-1.318,3.737]	1.091 [-4.374,6.676]	-0.235 [-3.131,2.651]
	-0.028 [-0.12,0.064]	1.266 [-0.142,0.101]	1.303 [-0.558,3.175]	4.465 [-4.906,13.806]	-0.456 [-1.137,0.231]	0.427 [-0.791,1.651]	1.589 [0.33,2.834]

Note: White rows correspond to the effects of social media use (vs non-use) and grey rows correspond to the effects of social media use duration. 95% Bayesian credible intervals are presented below each co-efficient in square brackets.

Table S16: Social Context Moderators (Momentary Feelings of Being Accepted) (Exploratory Sample)

	Alone	Family Ties	Close Ties	Distant Ties
Intercept	2.525 [2.469,2.579]	2.543 [2.487,2.595]	2.536 [0.275,0.778]	2.542 [2.486,2.598]
	2.512 [2.453,2.571]	0.136 [0.091,0.182]	0.23 [0.185,0.275]	0.032 [-0.021,0.083]
Context (WP)	-0.268 [-0.291,-0.246]	0.192 [0.164,0.22]	0.304 [0.275,0.332]	0.027 [0.002,0.051]
	-0.191 [-0.241,-0.142]	0.194 [0.113,0.276]	0.033 [-0.111,0.178]	-0.078 [-0.39,0.232]
Context (BP)	-0.926 [-1.138,-0.71]	-0.027 [-0.278,0.233]	0.586 [0.386,0.778]	0.379 [-0.13,0.877]
	-0.891 [-1.125,-0.666]	-0.028 [-0.285,0.231]	0.645 [0.439,0.852]	0.312 [-0.224,0.851]
Social Media Use (vs Non-Use) (WP) × Context (WP)	0.088 [0.054,0.122]	-0.053 [-0.097,-0.01]	-0.069 [-0.108,-0.029]	-0.003 [-0.06,0.056]
	0.014 [-0.027,0.054]	0.036 [-0.01,0.082]	-0.029 [-0.079,0.021]	-0.023 [-0.09,0.044]
Social Media Use (vs Non-Use) (WP) × Context (BP)	0.08 [0.015,0.146]	-0.013 [-0.084,0.057]	-0.039 [-0.097,0.021]	-0.21 [-0.37,-0.049]
	0.044 [-0.024,0.113]	-0.016 [-0.09,0.059]	-0.025 [-0.087,0.036]	-0.11 [-0.292,0.069]
Social Media Use (vs Non-Use) (BP) × Context (BP)	-0.562 [-1.654,0.514]	-0.64 [-1.884,0.592]	0.483 [-0.547,1.511]	1.749 [-1.129,4.543]
	-0.086 [-0.406,0.239]	0.63 [0.271,0.996]	-0.099 [-0.394,0.184]	-0.171 [-0.952,0.628]

Note: White rows correspond to the effects of social media use (vs non-use) and grey rows correspond to the effects of social media use duration. 95% Bayesian credible intervals are presented below each co-efficient in square brackets.

2. Confirmatory Findings: Dataset 2 from Spring 2021

2a. Main Effects of Social Media Use on Wellbeing

Figure S2: Social Media Sensitivity Across Social and Affective Wellbeing (Confirmatory Sample)

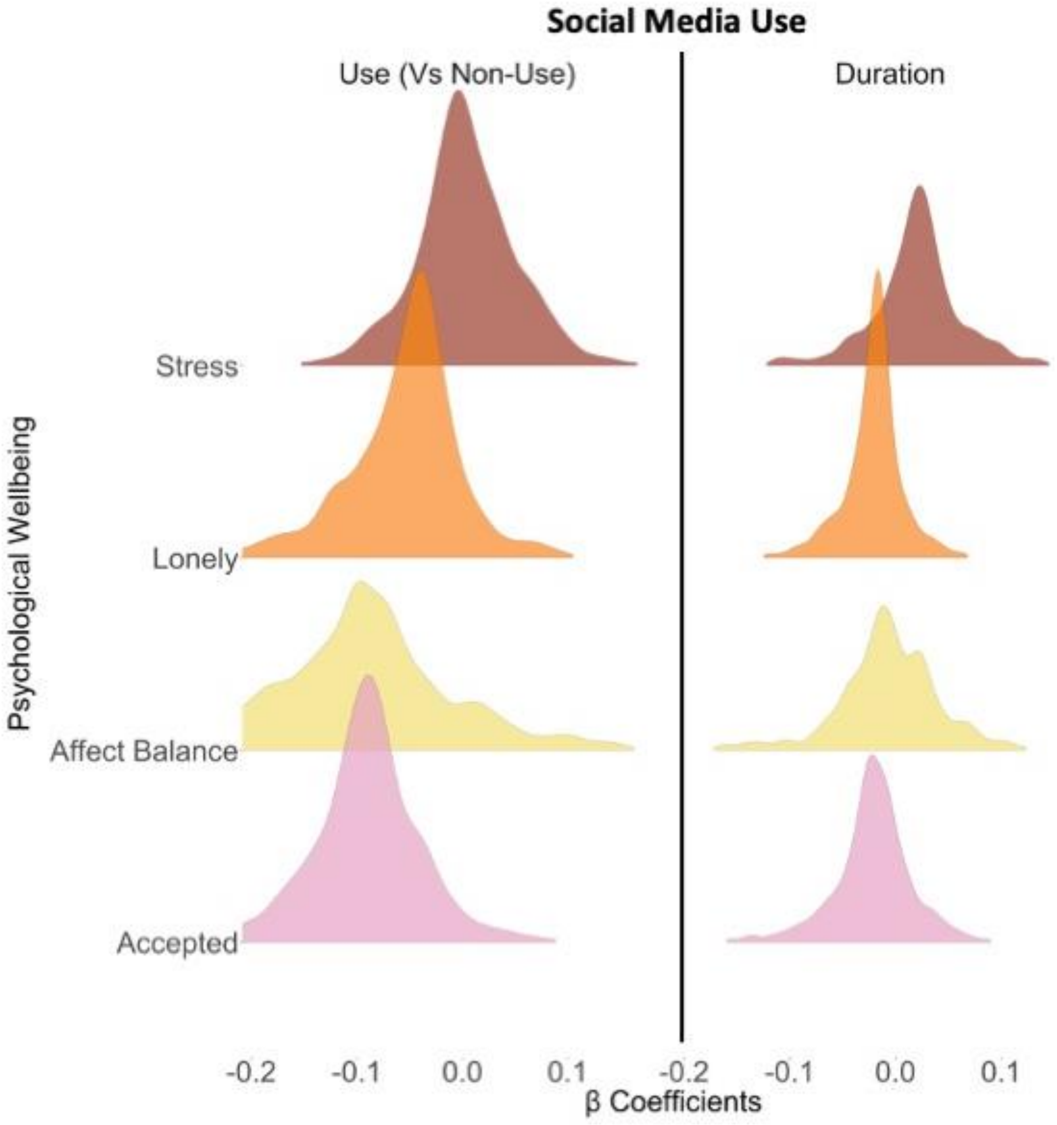

2b. Cross-Level Findings for Psychological Dispositions

Affect Balance. People who were higher in openness reported feeling a negative affect after using social media as compared to people who were lower in openness.
Lonely. People who had a dispositional negative affect balance reported feeling lonelier after using social media as compared to people who had a dispositional positive affect balance.

2c. Between-Person Findings for Psychological Dispositions

Stress. People who used social media for long durations of time and were high in openness generally reported greater stress as compared to people who used social media for long durations of time were low in openness.
Feelings of Being Accepted. People who used social media frequently and were higher in dispositional loneliness generally reported lower feelings of being accepted as compared to people who used social media frequently and were lower in dispositional loneliness. People who used social media for long durations of time and had a dispositional negative affect reported generally reported lower feelings of being accepted as compared to people who used social media for long durations of time and had a dispositional positive affect.

2d. Within-Person Findings for Context

Affect Balance. People reported a negative affect balance after using social media in nature as compared to after using social media in other types of places. People reported a negative affect balance after using social media around others as compared to after using social media alone. Specifically, people reported a negative affect balance after using social media around close ties as compared to other types of people.
Lonely. People reported greater feelings of loneliness after using social media around family ties as compared to after using social media around types of people.
Stress. People reported greater stress after using social media around close ties as compared to after using social media around other types of interaction partners. Similarly, people reported greater stress after using social media for greater durations than their own average when they were around family ties, as compared to when they were with other types of people. People reported greater stress after using social media than in nature, as compared to using social media in other types of places.
Accepted. People reported lower feelings of being accepted after using social media around others, as compared to after using social media when they were alone. People reported lower feelings of being accepted after using social media in nature as compared to after using social media in other types of places. People reported lower feelings of being accepted after using social media in social places, as compared to after using social media in other types of places. People reported lower feelings of being accepted after using social media in study places as compared to after using social media in other types of places.

2e. Cross-Level Findings for Context

Affect Balance. People who were in transit frequently reported a negative affect balance after using social media as compared to people who were not in transit frequently. People

who were around close ties often reported a negative affect balance after using social media for longer durations of time as compared to their own average, relative to people who were around close ties less often.
Accepted. People who were around close ties frequently reported lower feelings of being accepted after using social media as compared to people who were around close ties less frequently.

2f. Between-Person Findings for Context

Affect Balance. People who were in the gym frequently and reported using social media for long durations generally reported a negative affect balance as compared to people who were in the gym frequently and reported using social media for short durations of time. Similarly, people who were in transit frequently and reported using social media for long durations generally reported a negative affect balance as compared to people who were in transit less frequently and reported using social media for short durations.
Lonely. People who were around distant ties frequently and reported using social media for long durations of time generally reported higher feelings of loneliness as compared to people who were around distant ties less frequently. People who were in the gym frequently and reported using social media for greater durations of time reported feeling lonelier generally, as compared to people who were in the gym frequently and reported using social media for shorter durations of time. People who reported being outside the home frequently and used social media for long durations of time reported feeling lonelier on average, as compared to people who reported being outside the home frequently and used social media for shorter durations of time.
Stress. People who were around distant ties frequently and reported using social media for long durations of time generally reported higher feelings of stress, as compared to people who were around distant ties frequently and reported using social media for short of durations of time. People who were in social places frequently and reported using social media for long durations of time generally reported higher feelings of stress, as compared to people who were in social places frequently and reported using social media for short durations of time.
Accepted. People who were in the gym frequently and reported using social media more frequently reported generally greater feelings of being accepted as compared to people who were in the gym frequently and reported using social media less frequently.

Table S17: Dispositional Wellbeing Moderators (Momentary Loneliness) (Confirmatory Sample)

	**Affect Balance**	**Depression**	**Loneliness**	**Satisfaction with Life**
**Intercept**	3.455***	3.473***	3.455***	3.455***
	3.383***	3.395***	3.375***	3.383***
**Dispositional Trait**	0.142***	-0.023***	0.193**	0.025***
	0.149***	-0.033***	0.22**	0.022***
**Dispositional Trait (BP) x Social Media Use (BP)**	-0.064	0.01	0.201	-0.022
	0.018	0.004	-0.128	0.005
**Dispositional Trait (BP) x Social Media Use (WP)**	0.017**	-0.003T	0.025	0.002
	-0.003	0.001	0.008	0.000

Table S18: Big Five Trait Moderators (Momentary Loneliness) (Confirmatory Sample)

	**Openness**	**Conscientiousness**	**Extraversion**	**Agreeableness**	**Neuroticism**
**Intercept**	3.457***	3.456***	3.381***	3.461***	3.458***
	3.379***	3.378***	3.389***	3.385***	3.384***
**Dispositional Trait**	-0.056	0.123**	0.074*	0.2***	-0.239***
	-0.073T	0.126***	0.08**	0.206***	-0.257***
**Dispositional Trait (BP) x Social Media Use (WP)**	0.048	0.025	-0.022	0.359	0.039
	0.09	-0.006	0.012	0.032	-0.025
**Dispositional Trait (BP) x Social Media Use (BP)**	-0.026T	0.007	0.000	0.004	-0.022T
	0.001	-0.015	-0.008	0.003	-0.004

Table S19: Dispositional Wellbeing Moderators (Momentary Stress) (Confirmatory Sample)

	**Affect Balance**	**Depression**	**Loneliness**	**Satisfaction with Life**
**Intercept**	2.93***	2.942***	2.929***	2.93***
	2.891***	2.886***	2.891***	2.893***
**Dispositional Trait**	0.166***	-0.025***	0.133^{T}	0.024***
	0.173***	-0.032***	0.109	0.022***
**Dispositional Trait (BP) x Social Media Use (BP)**	-0.01	0.019	0.556	-0.015
	-0.017	0.013^{T}	-0.183	-0.005
**Dispositional Trait (BP) x Social Media Use (WP)**	0.002	-0.001	-0.003	0
	-0.002	0.002	0.024	0

Table S20: Big Five Trait Moderators (Momentary Stress) (Confirmatory Sample)

	Openness	Conscientiousness	Extraversion	Agreeableness	Neuroticism
**Intercept**	2.934***	2.933***	2.933***	2.938***	2.93***
	2.887***	2.888***	2.886***	2.893***	2.881***
**Dispositional Trait**	0.069T	0.123**	0.049	0.137**	-0.263***
	0.062	0.115***	0.038	0.119*	-0.256***
**Dispositional Trait (BP) x Social Media Use (BP)**	0.026	-0.097	0.241	-0.056	0.199
	0.143*	-0.001	0.029	0.061	0.045
**Dispositional Trait (BP) x Social Media Use (WP)**	-0.028T	0.007	-0.004	-0.026	-0.012
	-0.02	0.011	0.009	0.002	-0.01

Table S21: Dispositional Wellbeing Moderators (Momentary Affect Balance) (Confirmatory Sample)

	Affect Balance	Depression	Loneliness	Satisfaction with Life
**Intercept**	1.154***	1.164***	1.151***	1.153***
	1.07***	1.096***	1.075***	1.077***
**Dispositional Trait**	0.324***	-0.048***	0.629***	0.067***
	0.32***	-0.057***	0.543***	0.063***
**Dispositional Trait (BP) x Social Media Use (BP)**	0.095	0.012	0.961^{T}	0.003
	-0.025	0.003	0.005	0
**Dispositional Trait (BP) x Social Media Use (WP)**	0.014	-0.002	-0.058	0.002
	0	0.003	0.037	-0.002

Table S22: Big Five Trait Moderators (Momentary Affect Balance) (Confirmatory Sample)

	Openness	Conscientiousness	Extraversion	Agreeableness	Neuroticism
**Intercept**	1.16***	1.16***	1.163***	1.165***	1.158***
	1.073***	1.074***	1.077***	1.077***	1.074***
**Dispositional Trait**	0.062	0.342***	0.36***	0.453***	-0.552***
	0.045	0.328***	0.331***	0.383***	-0.529***
**Dispositional Trait (BP) x Social Media Use (BP)**	-0.091	-0.118	0.166	0.364	0.196
	0.095	0.005	-0.038	-0.037	0.007
**Dispositional Trait (BP) x Social Media Use (WP)**	-0.053*	0.006	-0.034	-0.04	-0.021
	-0.012	-0.025	0.003	-0.014	-0.004

Table S23: Dispositional Wellbeing Moderators (Momentary Feelings of Being Accepted) (Confirmatory Sample)

	Affect Balance	Depression	Loneliness	Satisfaction with Life
**Intercept**	2.618***	2.621	2.614***	2.618***
	2.565***	2.585***	2.574***	2.576***
**Dispositional Trait**	0.223***	-0.028***	0.747***	0.055***
	0.225***	-0.037***	0.749***	0.052***
**Dispositional Trait (BP) x Social Media Use (BP)**	0.08	0.016	1.229*	0.026
	-0.058*	0.002	-0.083	-0.006
**Dispositional Trait (BP) x Social Media Use (WP)**	0.011	-0.002	-0.002	-0.001
	-0.004	-0.002	0.041	0.002

Table S24: Big Five Trait Moderators (Momentary Feelings of Being Accepted) (Confirmatory Sample)

	Openness	Conscientiousness	Extraversion	Agreeableness	Neuroticism
**Intercept**	2.615***	2.614***	2.613***	2.612***	2.611***
	2.564	2.563	2.562***	2.558	2.566
**Dispositional Trait**	0.085	0.233***	0.385***	0.381***	-0.356***
	0.059	0.199	0.354***	0.333***	-0.335***
**Dispositional Trait (BP) x Social Media Use (BP)**	-0.013	-0.056	0.057	0.635*	0.167
	-0.041	-0.034	-0.042	-0.057	-0.033
**Dispositional Trait (BP) x Social Media Use (WP)**	-0.009	-0.01	-0.008	-0.02	-0.021
	0.013	-0.016	0.008	0.014	-0.019

Table S25: Physical Context Moderators (Momentary Loneliness) (Confirmatory Sample)

	Home	Nature	Gym	Religion	Social	Work	Transit	Study
**Intercept**	3.453 [3.408,3.501]	3.45 [3.404,3.497]	3.456 [3.411,3.501]	3.455 [3.408,3.502]	3.457 [3.408,3.506]	3.456 [3.41,3.503]	3.452 [3.405,3.498]	3.454 [3.405,3.5]
	3.384 [3.328,3.441]	3.389 [3.331,3.447]	3.383 [3.326,3.44]	3.378 [3.32,3.434]	3.38 [3.323,3.436]	3.375 [3.318,3.433]	3.375 [3.319,3.431]	3.375 [3.319,3.432]
**Context (WP)**	-0.085 [-0.105,-0.065]	0.075 [0.05,0.099]	0.049 [0.005,0.093]	-0.041 [-0.173,0.093]	0.122 [0.097,0.147]	-0.001 [-0.044,0.042]	0.072 [0.05,0.094]	-0.027 [-0.058,0.005]
	-0.077 [-0.126,-0.028]	0.062 [-0.011,0.135]	0.024 [-0.113,0.158]	-0.129 [-0.509,0.259]	0.081 [0.032,0.129]	-0.051 [-0.171,0.07]	0.082 [0.032,0.132]	-0.027 [-0.095,0.039]
**Context (BP)**	0.129 [-0.043,0.299]	0.585 [-0.191,1.371]	-0.339 [-1.485,0.811]	0.563 [-3.059,4.147]	-0.171 [-0.523,0.181]	0.331 [-0.34,0.993]	0.36 [-0.276,0.991]	-0.075 [-0.297,0.143]
	0.099 [-0.106,0.306]	0.641 [-0.254,1.511]	0.236 [-1.09,1.61]	1.754 [-2.098,5.567]	-0.121 [-0.521,0.275]	0.357 [-0.38,1.097]	-0.066 [-0.719,0.6]	-0.105 [-0.34,0.14]
**Social Media Use (vs Non-Use) (WP) x Context (WP)**	0.005 [-0.04,0.05]	0.005 [-0.065,0.075]	0.01 [-0.13,0.148]	-0.049 [-0.434,0.351]	-0.016 [-0.07,0.038]	0.008 [-0.127,0.144]	0.037 [-0.017,0.092]	0.032 [-0.03,0.095]
	-0.015 [-0.071,0.04]	0.023 [-0.067,0.114]	0.036 [-0.194,0.282]	0.126 [-0.769,0.899]	0.031 [-0.028,0.091]	-0.151 [-0.31,0.016]	-0.002 [-0.063,0.06]	0.034 [-0.04,0.108]
**Social Media Use (vs Non-Use) (WP) x Context (BP)**	0.028 [-0.043,0.099]	0.039 [-0.25,0.326]	-0.168 [-0.651,0.313]	0.127 [-1.323,1.568]	-0.087 [-0.23,0.054]	0.071 [-0.201,0.343]	-0.006 [-0.219,0.204]	0.005 [-0.082,0.091]
	0.02 [-0.045,0.086]	-0.097 [-0.366,0.175]	-0.361 [-0.859,0.134]	-0.219 [-1.99,1.496]	0.02 [-0.114,0.155]	-0.141 [-0.418,0.129]	-0.037 [-0.229,0.154]	-0.021 [-0.104,0.061]
**Social Media Use (vs Non-Use) (BP) x Context (BP)**	0.427 [-0.464,1.314]	2.07 [-1.298,5.382]	-2.411 [-9.736,4.875]	-19.586 [-45.805,7.08]	-0.814 [-2.634,0.965]	0.084 [-4.35,4.6]	0.562 [-2.006,3.16]	0.045 [-0.935,1.008]
	0.294 [0.019,0.565]	0.947 [-0.407,2.28]	-2.903 [-4.778,-0.999]	-2.791 [-7.248,1.769]	-0.908 [-1.483,-0.347]	-0.029 [-1.206,1.118]	-0.763 [-1.804,0.293]	-0.044 [-0.347,0.269]

Note: White rows correspond to the effects of social media use (vs non-use) and grey rows correspond to the effects of social media use duration. 95% Bayesian credible intervals are presented below each co-efficient in square brackets.

Table S26 – Social Context Moderators (Momentary Loneliness) (Confirmatory Sample)

	Alone	Family Ties	Close Ties	Distant Ties
**Intercept**	3.367 [3.31,3.424]	3.455 [3.408,0.13]	3.455 [3.408,3.501]	3.454 [3.407,3.502]
	3.373 [3.317,3.431]	3.373 [3.317,3.431]	3.374 [3.317,3.43]	3.375 [3.319,3.432]
**Context (WP)**	-0.008 [-0.045,0.029]	0.07 [0.05,0.089]	0.121 [0.1,0.141]	0.015 [-0.005,0.036]
	0.094 [0.058,0.13]	0.094 [0.058,0.13]	0.083 [0.04,0.125]	-0.015 [-0.072,0.041]
**Context (BP)**	0.024 [-0.06,0.106]	-0.05 [-0.26,0.156]	0.225 [0.05,0.402]	-0.082 [-0.496,0.323]
	-0.177 [-0.421,0.066]	-0.177 [-0.421,0.066]	0.267 [0.055,0.469]	-0.122 [-0.583,0.34]
**Social Media Use (vs Non-Use) (WP) x Context (WP)**	0.416 [0.104,0.72]	0.04 [0.003,0.076]	-0.024 [-0.067,0.018]	-0.035 [-0.097,0.028]
	0.023 [-0.015,0.062]	0.023 [-0.015,0.062]	-0.022 [-0.062,0.019]	-0.022 [-0.086,0.042]
**Social Media Use (vs Non-Use) (WP) x Context (BP)**	3.373 [3.317,3.431]	-0.005 [-0.092,0.08]	0.015 [-0.055,0.084]	0.046 [-0.119,0.213]
	0.075 [-0.008,0.156]	0.075 [-0.008,0.156]	-0.047 [-0.114,0.02]	-0.006 [-0.09,0.079]
**Social Media Use (vs Non-Use) (BP) x Context (BP)**	0.094 [0.058,0.13]	-0.602 [-1.875,0.659]	-0.239 [-1.143,0.64]	0.919 [-0.9,2.703]
	-0.356 [-0.691,-0.013]	-0.356 [-0.691,-0.013]	-0.12 [-0.431,0.191]	-0.772 [-1.468,-0.083]

Note: White rows correspond to the effects of social media use (vs non-use) and grey rows correspond to the effects of social media use duration. 95% Bayesian credible intervals are presented below each co-efficient in square brackets.

Table S27 – Physical Context Moderators (Momentary Stress) (Confirmatory Sample)

	Home	Nature	Gym	Religion	Social	Work	Transit	Study
**Intercept**	2.929 [2.879,2.981]	2.935 [2.882,2.989]	2.934 [2.882,2.986]	2.931 [2.879,2.984]	2.927 [2.875,2.98]	2.933 [2.883,2.985]	2.927 [2.875,2.979]	2.93 [2.88,2.98]
	2.896 [2.833,2.959]	2.901 [2.837,2.964]	2.899 [2.837,2.961]	2.895 [2.834,2.958]	2.9 [2.838,2.962]	2.892 [2.828,2.953]	2.902 [2.839,2.967]	2.89 [2.828,2.952]
**Context (WP)**	-0.089 [-0.112,-0.066]	0.158 [0.128,0.188]	0.085 [0.033,0.139]	0.012 [-0.128,0.151]	0.124 [0.097,0.15]	-0.006 [-0.054,0.042]	0.116 [0.09,0.141]	-0.117 [-0.15,-0.083]
	-0.042 [-0.094,0.009]	0.012 [-0.074,0.099]	-0.078 [-0.246,0.082]	-0.144 [-0.587,0.319]	0.083 [0.029,0.136]	-0.076 [-0.241,0.097]	0.119 [0.063,0.175]	-0.121 [-0.19,-0.051]
**Context (BP)**	0.191 [-0.02,0.393]	1.104 [0.235,1.97]	-0.285 [-1.549,0.971]	-0.379 [-4.329,3.587]	-0.415 [-0.835,0.007]	-0.312 [-1.061,0.418]	-0.319 [-1.045,0.394]	-0.025 [-0.272,0.222]
	0.117 [-0.106,0.336]	1.299 [0.334,2.28]	0.378 [-1.077,1.856]	-1.317 [-5.457,2.78]	-0.358 [-0.805,0.077]	-0.227 [-1.031,0.579]	-0.178 [-0.893,0.541]	0.052 [-0.207,0.313]
**Social Media Use (vs Non-Use) (WP) × Context (WP)**	0.03 [-0.02,0.08]	-0.166 [-0.251,-0.081]	-0.15 [-0.326,0.024]	-0.099 [-0.575,0.394]	-0.024 [-0.088,0.04]	0.002 [-0.128,0.003]	-0.025 [-0.091,0.042]	0.001 [-0.07,0.071]
	0.047 [-0.007,0.101]	-0.08 [-0.183,0.023]	-0.108 [-0.336,0.112]	0.315 [-0.661,1.197]	-0.012 [-0.08,0.057]	-0.144 [-0.378,0.09]	-0.009 [-0.08,0.065]	-0.031 [-0.12,0.06]
**Social Media Use (vs Non-Use) (WP) × Context (BP)**	-0.007 [-0.092,0.076]	-0.106 [-0.434,0.219]	-0.167 [-0.707,0.374]	-0.348 [-2.033,1.357]	-0.07 [-0.237,0.095]	0.001 [-0.114,0.506]	-0.028 [-0.265,0.209]	0.083 [-0.016,0.179]
	0.032 [-0.052,0.115]	-0.244 [-0.572,0.086]	-0.117 [-0.7,0.478]	0.998 [-1.043,3.036]	-0.116 [-0.274,0.042]	-0.038 [-0.373,0.297]	-0.119 [-0.344,0.109]	-0.034 [-0.134,0.067]
**Social Media Use (vs Non-Use) (BP) × Context (BP)**	0.07 [-0.059,0.197]	-1.553 [-5.213,2.155]	2.642 [-5.402,10.763]	-6.648 [-35.869,23.471]	0.862 [-1.234,2.989]	0.034 [-6.674,3.093]	1.019 [-1.845,3.977]	-0.155 [-1.194,0.918]
	0.002 [-0.081,0.084]	0.66 [-0.785,2.157]	-1.868 [-3.943,0.21]	-3.854 [-8.874,1.131]	-0.973 [-1.605,-0.348]	-0.042 [-1.322,1.23]	-0.042 [-1.212,1.122]	0.067 [-0.284,0.415]

Note: White rows correspond to the effects of social media use (vs non-use) and grey rows correspond to the effects of social media use duration. 95% Bayesian credible intervals are presented below each co-efficient in square brackets.

Table S28 – Social Context Moderators (Momentary Stress) (Confirmatory Sample)

	Alone	Family Ties	Close Ties	Distant Ties
**Intercept**	2.934 [2.883,2.986]	2.933 [2.881,2.985]	2.934 [0.042,0.084]	2.933 [2.88,2.985]
	2.893 [2.831,2.956]	2.892 [2.829,2.953]	2.901 [-0.039,0.072]	2.895 [2.833,2.958]
**Context (WP)**	-0.073 [-0.091,-0.056]	0.073 [0.052,0.094]	0.062 [0.042,0.083]	-0.007 [-0.031,0.017]
	0.022 [0.001,0.043]	0.089 [0.053,0.125]	0.005 [-0.039,0.05]	-0.012 [-0.071,0.048]
**Context (BP)**	0.158 [-0.081,0.384]	0.001 [-0.233,0.24]	-0.128 [-0.334,0.084]	-0.049 [-0.504,0.408]
	0.164 [-0.091,0.422]	-0.018 [-0.279,0.243]	-0.148 [-0.367,0.072]	0.004 [-0.489,0.506]
**Social Media Use (vs Non-Use) (WP) x Context (WP)**	0.034 [-0.006,0.075]	0.033 [-0.012,0.078]	-0.087 [-0.136,-0.038]	-0.009 [-0.076,0.058]
	0.02 [-0.02,0.059]	-0.064 [-0.107,-0.021]	0.019 [-0.028,0.067]	0.02 [-0.059,0.1]
**Social Media Use (vs Non-Use) (WP) x Context (BP)**	-0.028 [-0.122,0.067]	-0.007 [-0.104,0.09]	-0.004 [-0.084,0.078]	0.172 [-0.012,0.359]
	0.064 [-0.037,0.163]	-0.053 [-0.155,0.048]	-0.017 [-0.096,0.062]	-0.019 [-0.198,0.164]
**Social Media Use (vs Non-Use) (BP) x Context (BP)**	0.996 [-0.313,2.319]	-0.621 [-1.969,0.774]	-0.305 [-1.383,0.755]	0.461 [-1.555,2.477]
	0.275 [-0.065,0.615]	-0.292 [-0.664,0.077]	-0.078 [-0.421,0.269]	-0.791 [-1.534,-0.041]

Note: White rows correspond to the effects of social media use (vs non-use) and grey rows correspond to the effects of social media use duration. 95% Bayesian credible intervals are presented below each co-efficient in square brackets.

Table S29 – Physical Context Moderators (Momentary Affect Balance) (Confirmatory Sample)

	Home	Nature	Gym	Religion	Social	Work	Transit	Study
**Intercept**	1.149 [1.079,1.222]	1.142 [1.069,1.213]	1.156 [1.085,1.227]	1.152 [1.08,1.223]	1.146 [1.074,1.216]	1.154 [1.085,1.223]	1.145 [1.071,1.218]	1.152 [1.08,1.223]
	1.087 [1.001,1.172]	1.079 [0.992,1.166]	1.075 [0.992,1.16]	1.073 [0.987,1.158]	1.073 [0.988,1.157]	1.07 [0.985,1.156]	1.062 [0.975,1.147]	1.068 [0.982,1.154]
**Context (WP)**	-0.266 [-0.298,-0.234]	0.4 [0.354,0.446]	0.279 [0.195,0.363]	-0.001 [-0.231,0.227]	0.354 [0.313,0.396]	-0.08 [-0.155,-0.006]	0.174 [0.137,0.21]	-0.099 [-0.145,-0.051]
	-0.175 [-0.239,-0.112]	0.18 [0.058,0.304]	-0.009 [-0.204,0.186]	0.088 [-0.428,0.598]	0.242 [0.169,0.315]	-0.201 [-0.421,0.031]	0.107 [0.026,0.19]	-0.03 [-0.122,0.063]
**Context (BP)**	-0.027 [-0.316,0.256]	2.345 [1.186,3.52]	1.343 [-0.369,3.137]	-0.539 [-6.214,5.002]	0.331 [-0.242,0.903]	-0.212 [-1.22,0.81]	1.055 [0.102,2.062]	0.031 [-0.313,0.375]
	-0.099 [-0.399,0.207]	2.512 [1.183,3.889]	2.049 [0.011,4.08]	-0.712 [-6.334,4.906]	0.395 [-0.207,0.999]	-0.216 [-1.346,0.874]	0.298 [-0.671,1.255]	0.011 [-0.35,0.37]
**Social Media Use (vs Non-Use) (WP) × Context (WP)**	0.052 [-0.019,0.124]	-0.142 [-0.26,-0.023]	-0.243 [-0.486,0.003]	0.287 [-0.334,0.951]	-0.077 [-0.165,0.011]	-0.027 [-0.231,0.178]	-0.024 [-0.116,0.07]	0.079 [-0.019,0.178]
	0.066 [-0.011,0.14]	-0.114 [-0.261,0.035]	-0.201 [-0.468,0.067]	-0.726 [-1.659,0.207]	-0.087 [-0.189,0.016]	0.001 [-0.001,0.004]	-0.041 [-0.153,0.07]	-0.041 [-0.147,0.065]
**Social Media Use (vs Non-Use) (WP) × Context (BP)**	0.088 [-0.037,0.211]	-0.323 [-0.798,0.152]	0.06 [-0.737,0.857]	-0.939 [-3.41,1.558]	-0.167 [-0.411,0.077]	0.034 [-0.421,0.485]	-0.37 [-0.725,-0.018]	0.051 [-0.095,0.197]
	0.047 [-0.065,0.159]	-0.161 [-0.596,0.272]	-0.292 [-1.063,0.462]	0.402 [-2.136,2.944]	-0.106 [-0.319,0.108]	-0.001 [-0.002,0.001]	-0.069 [-0.369,0.231]	-0.065 [-0.196,0.066]
**Social Media Use (vs Non-Use) (BP) × Context (BP)**	0.177 [-0.004,0.362]	4.533 [-0.609,9.567]	8.682 [-2.427,19.681]	-21.314 [-61.654,18.996]	1.032 [-1.777,3.792]	-4.353 [-10.918,2.331]	1.631 [-2.403,5.636]	0.057 [-1.412,1.505]
	0.014 [-0.088,0.117]	0.34 [-0.299,0.039]	-1.772 [-4.607,1.031]	-2.697 [-9.51,3.984]	-0.984 [-1.834,-0.12]	-0.006 [-0.267,0.252]	-1.789 [-3.358,-0.22]	-0.119 [-0.588,0.35]

Note: White rows correspond to the effects of social media use (vs non-use) and grey rows correspond to the effects of social media use duration. 95% Bayesian credible intervals are presented below each co-efficient in square brackets.

Table S30– Social Context Moderators (Momentary Affect Balance) (Confirmatory Sample)

	Alone	Family Ties	Close Ties	Distant Ties
**Intercept**	1.127 [1.056,1.198]	1.156 [1.085,1.228]	1.144 [0.253,1.045]	1.152 [1.079,1.223]
	1.063 [0.978,1.15]	0.121 [0.07,0.172]	0.165 [0.102,0.227]	0.033 [-0.059,0.125]
**Context (WP)**	-0.275 [-0.301,-0.248]	0.148 [0.119,0.177]	0.287 [0.253,0.32]	0.083 [0.047,0.118]
	-0.175 [-0.239,-0.112]	0.18 [0.058,0.304]	-0.009 [-0.204,0.186]	0.088 [-0.428,0.598]
**Context (BP)**	-0.852 [-1.171,-0.528]	-0.028 [-0.345,0.292]	0.77 [0.486,1.045]	0.575 [-0.049,1.18]
	-0.661 [-1.015,-0.306]	-0.169 [-0.536,0.194]	0.687 [0.39,0.98]	0.446 [-0.256,1.131]
**Social Media Use (vs Non-Use) (WP) × Context (WP)**	0.103 [0.043,0.163]	-0.021 [-0.083,0.043]	-0.117 [-0.184,-0.051]	-0.023 [-0.117,0.073]
	0.058 [-0.001,0.118]	-0.033 [-0.092,0.026]	-0.021 [-0.092,0.05]	-0.056 [-0.167,0.058]
**Social Media Use (vs Non-Use) (WP) × Context (BP)**	0.089 [-0.05,0.227]	-0.052 [-0.195,0.091]	-0.038 [-0.157,0.082]	-0.04 [-0.318,0.236]
	0.123 [-0.01,0.255]	0.065 [-0.067,0.195]	-0.128 [-0.231,-0.024]	-0.056 [-0.291,0.183]
**Social Media Use (vs Non-Use) (BP) × Context (BP)**	-0.583 [-2.383,1.242]	-0.871 [-2.805,1.005]	0.037 [-1.374,1.458]	1.434 [-1.286,4.202]
	0.353 [-0.104,0.806]	-0.304 [-0.803,0.204]	-0.101 [-0.555,0.357]	-0.854 [-1.884,0.156]

Note: White rows correspond to the effects of social media use (vs non-use) and grey rows correspond to the effects of social media use duration. 95% Bayesian credible intervals are presented below each co-efficient in square brackets.

Table S31 – Physical Context Moderators (Momentary Feelings of Being Accepted) (Confirmatory Sample)

	Home	Nature	Gym	Religion	Social	Work	Transit	Study
**Intercept**	2.615 [2.553,2.676]	2.602 [2.54,2.664]	2.618 [2.555,2.678]	2.614 [2.552,2.676]	2.613 [2.55,2.675]	2.615 [2.554,2.679]	2.608 [2.542,2.672]	2.617 [2.554,2.681]
	2.586 [2.513,2.656]	2.576 [2.504,2.647]	2.576 [2.503,2.648]	2.578 [2.507,2.65]	2.574 [2.504,2.644]	2.573 [2.499,2.645]	2.567 [2.495,2.64]	2.577 [2.505,2.65]
**Context (WP)**	-0.183 [-0.208,-0.159]	0.186 [0.151,0.22]	0.127 [0.071,0.182]	0.142 [-0.014,0.296]	0.256 [0.224,0.287]	0.062 [0.001,0.123]	0.134 [0.107,0.162]	-0.058 [-0.091,-0.025]
	-0.106 [-0.149,-0.063]	0.077 [-0.001,0.157]	0.055 [-0.095,0.209]	0.169 [-0.256,0.614]	0.155 [0.095,0.215]	-0.015 [-0.151,0.125]	0.084 [0.029,0.141]	0.033 [-0.026,0.094]
**Context (BP)**	-0.208 [-0.447,0.045]	1.909 [0.871,2.933]	1.963 [0.396,3.542]	0.566 [-4.354,5.765]	0.717 [0.228,1.217]	-0.562 [-1.44,0.343]	1.037 [0.2,1.94]	0.127 [-0.174,0.421]
	-0.282 [-0.548,-0.016]	2.434 [1.283,3.585]	2.478 [0.725,4.205]	-0.264 [-5.224,4.588]	0.909 [0.399,1.428]	-0.788 [-1.753,0.149]	0.671 [-0.171,1.531]	0.124 [-0.191,0.435]
**Social Media Use (vs Non-Use) (WP) × Context (WP)**	0.04 [-0.008,0.089]	-0.086 [-0.17,-0.004]	-0.027 [-0.191,0.139]	0.227 [-0.258,0.747]	-0.077 [-0.14,-0.014]	0.054 [-0.093,0.202]	-0.001 [-0.065,0.064]	0.114 [0.043,0.186]
	0.021 [-0.033,0.074]	-0.039 [-0.14,0.062]	0.068 [-0.146,0.272]	-0.25 [-1.123,0.769]	-0.025 [-0.095,0.044]	-0.152 [-0.349,0.038]	-0.053 [-0.124,0.018]	-0.002 [-0.078,0.074]
**Social Media Use (vs Non-Use) (WP) × Context (BP)**	0.017 [-0.065,0.099]	0.129 [-0.194,0.447]	0.262 [-0.276,0.795]	-1.366 [-3.056,0.364]	-0.001 [-0.162,0.161]	0.085 [-0.226,0.393]	-0.121 [-0.363,0.116]	0.052 [-0.047,0.151]
	0.061 [-0.016,0.138]	-0.04 [-0.347,0.271]	0.035 [-0.509,0.579]	2.43 [0.532,4.352]	-0.076 [-0.22,0.07]	-0.198 [-0.507,0.108]	-0.062 [-0.279,0.155]	-0.018 [-0.113,0.075]
**Social Media Use (vs Non-Use) (BP) × Context (BP)**	0.171 [0.028,0.313]	5.257 [-0.19,0.071]	11.693 [1.864,21.534]	-4.394 [-40.023,31.129]	1.073 [-1.462,3.547]	-3.265 [-9.348,2.757]	2.03 [-1.543,5.675]	-0.062 [-1.385,1.261]
	-0.036 [-0.105,0.034]	-0.131 [-0.232,0.062]	-0.074 [-2.577,2.376]	0.481 [-5.405,6.273]	-0.517 [-1.25,0.215]	-0.069 [-1.584,1.446]	-1.326 [-2.734,0.074]	-0.168 [-0.573,0.225]

Note: White rows correspond to the effects of social media use (vs non-use) and grey rows correspond to the effects of social media use duration. 95% Bayesian credible intervals are presented below each co-efficient in square brackets.

Table S32 – Social Context Moderators (Momentary Feelings of Being Accepted) (Confirmatory Sample)

	Alone	Family Ties	Close Ties	Distant Ties
**Intercept**	2.591 [2.529,2.652]	2.619 [2.554,2.684]	2.615 [0.233,1.157]	2.612 [2.548,2.678]
	2.559 [2.489,2.63]	0.109 [0.071,0.147]	0.21 [0.166,0.256]	0.072 [0.01,0.134]
**Context (WP)**	-0.263 [-0.287,-0.24]	0.135 [0.112,0.157]	0.261 [0.233,0.289]	0.077 [0.051,0.104]
	-0.175 [-0.239,-0.112]	0.18 [0.058,0.304]	-0.009 [-0.204,0.186]	0.088 [-0.428,0.598]
**Context (BP)**	-1.21 [-1.479,-0.935]	0.091 [-0.19,0.389]	0.922 [0.693,1.157]	0.732 [0.188,1.291]
	-1.088 [-1.383,-0.79]	-0.037 [-0.358,0.279]	0.918 [0.668,1.176]	0.631 [0.011,1.237]
**Social Media Use (vs Non-Use) (WP) × Context (WP)**	0.051 [0.011,0.091]	-0.014 [-0.061,0.031]	-0.019 [-0.062,0.024]	0.011 [-0.06,0.082]
	0.036 [-0.007,0.079]	0 [-0.046,0.045]	-0.03 [-0.081,0.021]	0.018 [-0.059,0.097]
**Social Media Use (vs Non-Use) (WP) × Context (BP)**	0.062 [-0.028,0.152]	-0.032 [-0.128,0.065]	-0.023 [-0.101,0.055]	0.11 [-0.08,0.298]
	0.091 [-0.003,0.185]	0.063 [-0.034,0.158]	-0.078 [-0.15,-0.005]	0.018 [-0.154,0.194]
**Social Media Use (vs Non-Use) (BP) × Context (BP)**	-0.48 [-2.051,1.103]	-0.609 [-2.323,1.116]	-0.142 [-1.376,1.077]	1.532 [-0.969,3.953]
	0.377 [-0.012,0.766]	-0.191 [-0.627,0.24]	-0.143 [-0.527,0.251]	-0.006 [-0.878,0.872]

Note: White rows correspond to the effects of social media use (vs non-use) and grey rows correspond to the effects of social media use duration. 95% Bayesian credible intervals are presented below each co-efficient in square brackets.

3. Mega-analytic Findings: Exploratory and Confirmatory Datasets Merged

3a. Between-Person Findings for Psychological Dispositions

Loneliness. Frequent social media users who were higher in dispositional loneliness tended to report greater feelings of loneliness on average as compared to frequent social media users who were lower in dispositional loneliness.
Stress. Frequent social media users who were higher in dispositional loneliness tended to report greater feelings of stress generally, as compared to frequent social media users who were lower in dispositional loneliness. Similarly, people who tended to use social media for long durations of time and were higher in dispositional loneliness tended to report greater feelings of stress as compared to people who tended to use social media for long durations of time and were lower in dispositional loneliness.
Feelings of Being Accepted. Frequent social media users who were higher in depression, dispositional loneliness and lower in agreeableness tended to generally report lowers feelings of being accepted, as compared to frequent social media users who were lower in depression, dispositional loneliness and lower in agreeableness. Similarly, people who used social media for long durations of time and were higher in depression tended to report lowers feelings of being accepted as compared to people who used social media for longer durations of time and were lower in depression.

3b. Cross-Level Findings for Physical and Social Context

Loneliness. People who tended to be in transit contexts frequently reported feeling greater loneliness after using social media for longer durations than their own average, as compared to people who tended to be in transit context infrequently. Similarly, people who tended to be around distant ties frequently reported greater feelings of loneliness after using social media for longer durations of time than their own average as compared to people who tended to be around distant ties less frequently.
Stress. People who tended to be in nature contexts frequently reported feeling greater stress after using social media for long durations of time than their own average as compared to people who tended to be in nature contexts less frequently.
Affect Balance. People who tended to be in work contexts frequently reported a negative affect after using social media for longer durations than their own average, as compared to people who tended to be in work contexts less frequently.
Feelings of Being Accepted. People who tended to be in social places, workplaces and in transit frequently reported lower feelings of being accepted after using social media for longer durations than their own average as compared to people who tended to be in these locations less frequently. Similarly, people who tended to be in transit locations frequently reported a lower feeling of being accepted after using social media as compared to people who tended to be in transit less frequently. People who tended to be alone frequently reported greater feelings of being accepted after using social media platforms, as compared to people who tended to be alone less frequently. Conversely, people who tended to be with distant ties more frequently reported lower feelings of being accepted after using social media platforms, as compared to people who tended to be with distant ties less frequently.

3c. Between-Person Findings for Physical and Social Context

Loneliness. People who tended to use social media for long durations of time and tended to be at home infrequently reported lower feelings of loneliness generally, as compared to people who tended to use social media for long durations of time but tended to be at home frequently. Similarly, people who tended to use social media for long durations of time and tended to be at social places more frequently, reported higher feelings of loneliness generally, as compared to people who tended to use social media for long durations of time but tended to be at social places infrequently.
Stress. People who tended to use social media for long durations of time and tended to be in social places frequently reported higher feelings of stress generally, as compared to people who tended to use social media for long durations of time and but tended to be at social places infrequently. Similarly, people who tended to use social media for long durations of time and tended to be around distant ties frequently reported higher feelings of stress as compared to people who tended to use social media for long durations of time but tended to be around distant ties less frequently.
Affect Balance. People who tended to use social media for long durations of time and tended to be at social places frequently reported a negative affect generally, as compared to people who tended to use social media for long durations of time and tended to be at social places infrequently.
Feelings of Being Accepted. People who tended to use social media frequently and tended to be at the gym infrequently reported a positive affect as compared to people who tended to use social media frequently and tended to be at the gym frequently.

Table S33: Dispositional Moderators (Momentary Loneliness) (Mega-analysis)

	Openness	Conscientiousness	Extraversion	Agreeableness	Neuroticism	Affect Balance	Depression	Loneliness	Satisfaction with Life
**Intercept**	3.464***	3.465***	3.465***	3.463***	3.460***	3.453***	3.474***	3.366***	3.454***
	3.386***	3.390***	3.385***	3.384***	3.388***	3.373***	3.382***	3.276***	3.373***
**Dispositional Trait**	-0.032	0.080**	0.070**	0.134***	-0.235***	0.145***	-0.032***	-0.239***	0.026***
	-0.028	0.072*	0.080**	0.145***	-0.259***	0.150***	-0.038***	-0.249***	0.025***
**Sample**	-0.004	-0.008	-0.001	0.003	0.011	0.008	0.004	0.205***	0.003
	0.002	-0.004	0.007	0.009	0.015	0.014	0.029	0.223***	0.014
**Dispositional Trait (BP) x Social Media Use (WP)**	-0.004	0.006	0.012	0.007	-0.022**	0.015***	-0.003**	0.001	0.002*
	0.000	-0.004	-0.008	-0.012	0.002	-0.003	0.001	0.017^{T}	-0.001
**Dispositional Trait (BP) x Social Media Use (BP)**	0.030	-0.023	0.095	0.194	-0.151	-0.061	0.010	0.142	-0.016
	0.021	0.027	0.012	0.017	-0.058^{T}	0.019	-0.004	-0.087*	0.006^{T}

Note: White rows correspond to the effects of social media use (vs non-use) and grey rows correspond to the effects of social media use duration. ***p<0.001, **p<0.01, *p<0.05,^{T} p<0.1

Table S34: Dispositional Moderators (Momentary Stress) (Mega-analysis)

	**Openness**	**Conscientiousness**	**Extraversion**	**Agreeableness**	**Neuroticism**	**Affect Balance**	**Depression**	**Loneliness**	**Satisfaction with Life**
**Intercept**	2.790***	2.792***	2.792***	2.789***	2.780***	2.792***	2.790***	2.717***	2.793***
	2.785***	2.789***	2.788***	2.787***	2.783***	2.789***	2.777***	2.726***	2.790***
**Dispositional Trait**	0.007	0.073**	0.055*	0.101***	-0.278***	0.169***	-0.031***	-0.203***	0.028***
	-0.014	0.056 ^{T}	0.047^{T}	0.082*	-0.279***	0.167***	-0.037***	-0.171***	0.025***
**Sample**	0.144***	0.138***	0.141***	0.151***	0.158***	0.137***	0.154***	0.306***	0.132***
	0.126**	0.120**	0.124**	0.133***	0.137***	0.115**	0.143***	0.258***	0.116**
**Dispositional Trait (BP) x Social Media Use (WP)**	-0.018 ^{T}	-0.010	-0.014	-0.014	-0.009	-0.002	-0.001	0.021*	-0.001
	-0.014	0.016	0.003	-0.006	-0.007	-0.001	0.001	0.018 ^{T}	-0.001
**Dispositional Trait (BP) x Social Media Use (BP)**	-0.089	0.072	0.184	-0.247	0.097	-0.058	0.015	0.379*	-0.025 ^{T}
	0.050	0.024	0.048	0.085^{T}	-0.029	-0.003	-0.003	-0.101*	0.002

Note: White rows correspond to the effects of social media use (vs non-use) and grey rows correspond to the effects of social media use duration. ***p<0.001, **p<0.01, *p<0.05,^{T} p<0.1

Table S35: Dispositional Moderators (Momentary Affect Balance) (Mega-analysis)

	Openness	Conscientiousness	Extraversion	Agreeableness	Neuroticism	Affect Balance	Depression	Loneliness	Satisfaction with Life
**Intercept**	0.967***	0.970***	0.967***	0.964***	0.952***	0.975***	0.966***	0.813***	0.979***
	0.949***	0.957***	0.947***	0.950***	0.943***	0.946***	0.943***	0.784***	0.952***
**Dispositional Trait**	0.049	0.275***	0.374***	0.375***	-0.535***	0.338***	-0.064***	-0.438***	0.069***
	0.055	0.255***	0.360***	0.312***	-0.530***	0.335***	-0.070***	-0.425***	0.068***
**Sample**	0.200***	0.186***	0.203***	0.214***	0.230***	0.186***	0.217***	0.553***	0.174***
	0.163**	0.146**	0.171***	0.174***	0.190***	0.160***	0.199***	0.527***	0.156***
**Dispositional Trait (BP) x Social Media Use (WP)**	-0.019	-0.010	-0.016	-0.037*	-0.019	0.010	-0.004*	-0.012	0.001
	0.000	-0.003	0.004	-0.023	-0.006	0.001	0.001	0.020	-0.002
**Dispositional Trait (BP) x Social Media Use (BP)**	0.128	-0.041	0.215	0.263	-0.045	0.028	0.026^T	0.338^T	-0.017
	0.048	0.039	-0.011	0.038	-0.050	-0.012	-0.008	-0.023	0.003

Note: White rows correspond to the effects of social media use (vs non-use) and grey rows correspond to the effects of social media use duration. ***p<0.001, **p<0.01, *p<0.05, ^{T} p<0.1

Table S36: Dispositional Moderators (Momentary Feelings of Being Accepted) (Mega-analysis)

	Openness	Conscientiousness	Extraversion	Agreeableness	Neuroticism	Affect Balance	Depression	Loneliness	Satisfaction with Life
**Intercept**	2.515***	2.519***	2.513***	2.515***	2.503***	2.527***	2.517***	2.401***	2.530***
	2.485***	2.496***	2.482***	2.484***	2.482***	2.498***	2.496***	2.378***	2.502***
**Dispositional Trait**	0.110***	0.225***	0.388***	0.345***	-0.351***	0.221***	-0.041***	-0.341***	0.053***
	0.119***	0.185***	0.365***	0.307***	-0.342***	0.219***	-0.046***	-0.327***	0.050***
**Sample**	0.113**	0.102**	0.116**	0.119**	0.132**	0.103**	0.123**	0.390***	0.094*
	0.100***	0.086 ^{T}	0.104*	0.104*	0.116**	0.088*	0.112**	0.367***	0.088*
**Dispositional Trait (BP) x Social Media Use (WP)**	0.001	-0.015	0.001	-0.016	-0.012	0.006	-0.002	-0.013	0.000
	-0.004	-0.009	0.001	-0.005	-0.012	0.002	-0.002	0.005	0.000
**Dispositional Trait (BP) x Social Media Use (BP)**	0.101	-0.121	0.105	0.514*	0.021	0.026	0.033*	0.373*	-0.008
	-0.038	0.079	-0.018	0.013	-0.056	-0.016	-0.010*	-0.046	0.003

Note: White rows correspond to the effects of social media use (vs non-use) and grey rows correspond to the effects of social media use duration. ***p<0.001, **p<0.01, *p<0.05, ^{T} p<0.1

Table S37: Context Moderators (Momentary Loneliness) (Mega-analysis)

	Home	Nature	Gym	Religion	Social	Work	Transit	Study	Alone	Family Ties	Close Ties	Distant Ties
**Intercept**	3.456<br>[3.415,3.496]	3.459<br>[3.419,3.501]	3.456<br>[3.413,3.497]	3.458<br>[3.417,3.499]	3.459<br>[3.419,3.500]	3.453<br>[3.413,3.493]	3.460<br>[3.420,3.500]	3.455<br>[3.415,3.496]	3.462<br>[3.421,3.502]	3.456<br>[3.414,3.498]	3.462<br>[3.422,3.501]	3.453<br>[3.411,3.497]
	3.381<br>[3.332,3.428]	3.383<br>[3.332,3.432]	3.371<br>[3.323,3.419]	3.374<br>[3.325,3.422]	3.371<br>[3.321,3.420]	3.372<br>[3.323,3.420]	3.377<br>[3.329,3.425]	3.375<br>[3.326,3.423]	3.378<br>[3.329,3.426]	3.365<br>[3.316,3.415]	3.374<br>[3.325,3.422]	3.372<br>[3.324,3.421]
**Context (WP)**	-0.089<br>[-0.103,-0.075]	0.07<br>[0.052,0.088]	0.028<br>[-0.003,0.059]	-0.061<br>[-0.151,0.028]	0.121<br>[0.104,0.138]	0.018<br>[-0.011,0.048]	0.060<br>[0.045,0.076]	-0.034<br>[-0.056,-0.012]	-0.129<br>[-0.142,-0.116]	0.073<br>[0.060,0.087]	0.133<br>[0.118,0.148]	0.015<br>[0.002,0.029]
	-0.080<br>[-0.114,-0.045]	0.029<br>[-0.023,0.081]	-0.041<br>[-0.157,0.074]	-0.094<br>[-0.327,0.141]	0.093<br>[0.056,0.130]	0.004<br>[-0.074,0.082]	0.086<br>[0.051,0.120]	-0.042<br>[-0.090,0.006]	0.133<br>[0.156,0.109]	0.080<br>[0.056,0.103]	0.123<br>[0.095,0.151]	-0.002<br>[-0.041,0.036]
**Context (BP)**	0.092<br>[-0.026,0.205]	0.692<br>[0.133,1.262]	0.137<br>[-0.736,0.981]	1.512<br>[-1.148,4.238]	-0.116<br>[-0.341,0.113]	0.358<br>[-0.099,0.811]	0.496<br>[0.062,0.955]	-0.074<br>[-0.217,0.063]	-0.329<br>[-0.459,-0.196]	-0.004<br>[-0.136,0.133]	0.212<br>[0.095,0.327]	0.037<br>[-0.256,0.312]
	-0.004<br>[-0.131,0.126]	0.628<br>[-0.061,1.321]	0.228<br>[-0.724,1.175]	1.670<br>[-1.254,4.616]	-0.115<br>[-0.382,0.153]	0.378<br>[-0.117,0.873]	0.213<br>[-0.265,0.694]	-0.083<br>[-0.234,0.070]	-0.0314<br>[-0.467,0.163]	-0.038<br>[-0.201,0.121]	0.238<br>[0.104,0.370]	-0.073<br>[-0.400,0.250]
**Sample**	0.004<br>[-0.057,0.061]	-0.006<br>[-0.067,0.054]	0.004<br>[-0.056,0.065]	0.002<br>[-0.057,0.060]	0.005<br>[-0.053,0.063]	0.007<br>[-0.052,0.065]	0.001<br>[-0.058,0.057]	0.004<br>[-0.056,0.064]	-0.030<br>[-0.087,0.029]	0.002<br>[-0.060,0.064]	-0.004<br>[-0.059,0.052]	0.007<br>[-0.054,0.069]
	0.014<br>[-0.056,0.082]	0.003<br>[-0.067,0.073]	0.014<br>[-0.055,0.085]	0.011<br>[-0.057,0.079]	0.016<br>[-0.050,0.083]	0.012<br>[-0.056,0.079]	0.006<br>[-0.064,0.074]	0.017<br>[-0.050,0.085]	-0.014<br>[-0.083,0.055]	0.020<br>[-0.052,0.090]	0.006<br>[-0.063,0.075]	0.012<br>[-0.056,0.078]
**Social Media Use (WP) x Context (WP)**	-0.006<br>[-0.039,0.028]	-0.009<br>[-0.058,0.040]	-0.047<br>[-0.149,0.054]	0.036<br>[-0.195,0.272]	-0.017<br>[-0.053,0.020]	0.006<br>[-0.083,0.094]	0.041<br>[0.003,0.080]	0.038<br>[-0.005,0.080]	-0.012<br>[-0.036,0.011]	0.017<br>[-0.009,0.043]	0.010<br>[-0.016,0.036]	-0.010<br>[-0.050,0.031]
	0.009<br>[-0.029,0.047]	-0.043<br>[-0.112,0.026]	-0.031<br>[-0.188,0.125]	0.105<br>[-0.277,0.484]	0.000<br>[-0.042,0.041]	-0.125<br>[-0.225,-0.027]	0.007<br>[-0.038,0.051]	0.019<br>[-0.036,0.075]	-0.008<br>[-0.034,0.017]	0.024<br>[-0.005,0.052]	-0.022<br>[-0.052,0.008]	-0.012<br>[-0.055,0.032]
**Social Media Use (WP) x Context (BP)**	0.005<br>[-0.038,0.046]	0.021<br>[-0.169,0.212]	0.038<br>[-0.293,0.370]	0.053<br>[-0.998,1.083]	-0.045<br>[-0.134,0.044]	-0.112<br>[-0.288,0.063]	0.030<br>[-0.115,0.178]	0.024<br>[-0.025,0.074]	-0.044<br>[-0.092,0.005]	-0.015<br>[-0.035,0.065]	0.025<br>[-0.017,0.067]	-0.075<br>[-0.184,0.036]
	0.028<br>[-0.014,0.069]	-0.200<br>[-0.396,0.006]	-0.154<br>[-0.530,0.220]	-0.510<br>[-1.710,0.681]	-0.020<br>[-0.107,0.067]	-0.143<br>[-0.327,0.042]	-0.16 2<br>[-0.296,-0.026]	-0.032<br>[-0.082,0.017]	0.015<br>[-0.034,0.064]	0.030<br>[-0.021,0.082]	-0.014<br>[-0.056,0.028]	-0.112<br>[-0.223,-0.003]
**Social Media Use (BP) x Context (BP)**	0.412<br>[-0.222,1.065]	0.094<br>[-2.090,2.344]	-1.829<br>[-7.247,3.592]	-7.772<br>[-22.972,7.269]	-1.27<br>[-2.443,0.146]	-0.627<br>[-3.787,2.473]	-0.687<br>[-2.350,0.973]	0.113<br>[-0.586,0.815]	-0.228<br>[-0.898,0.461]	0.233<br>[-0.508,0.974]	-0.185<br>[-0.786,0.414]	0.113<br>[-1.325,1.510]
	0.199<br>[0.016,0.378]	0.694<br>[-0.299,1.667]	-0.923<br>[-2.121,0.262]	-0.953<br>[-4.915,3.059]	-0.713<br>[-1.119,-0.309]	-0.037<br>[-0.795,0.717]	-0.210<br>[-0.952,0.537]	-0.019<br>[-0.225,0.189]	0.175<br>[-0.033,0.380]	-0.188<br>[-0.412,0.033]	0.033<br>[-0.161,0.230]	-0.187<br>[-0.661,0.288]

Note: White rows correspond to the effects of social media use (vs non-use) and grey rows correspond to the effects of social media use duration. 95% Bayesian credible intervals are presented below each co-efficient in square brackets.

Table S38: Context Moderators (Momentary Stress) (Mega-analysis)

	Home	Nature	Gym	Religion	Social	Work	Transit	Study	Alone	Family Ties	Close Ties	Distant Ties
**Intercept**	2.788 [2.743,2.834]	2.802 [2.754,2.851]	2.795 [2.748,2.841]	2.792 [2.746,2.838]	2.791 [2.745,2.839]	2.793 [2.746,2.837]	2.794 [2.748,2.841]	2.791 [2.746,2.837]	2.786 [2.737,2.833]	2.789 [2.742,2.834]	2.796 [2.749,2.841]	2.792 [2.747,2.836]
	2.788 [2.736,2.840]	2.800 [2.746,2.853]	2.791 [2.738,2.844]	2.790 [2.738,2.841]	2.778 [2.735,2.838]	2.791 [2.740,2.843]	2.788 [2.735,2.841]	2.888 [2.736,2.840]	2.784 [2.732,2.837]	2.786 [2.733,2.838]	2.790 [0.738,0.842]	2.786 [2.734,2.838]
**Context (WP)**	-0.093 [-0.108,0.078]	0.137 [0.116,0.158]	0.113 [0.076,0.149]	0.055 [-0.033,0.143]	0.116 [0.098,0.133]	0.014 [-0.019,0.047]	0.102 [0.084,0.120]	-0.079 [-0.101,-0.056]	-0.061 [-0.072,-0.050]	0.047 [0.033,0.061]	0.070 [0.057,0.083]	-0.004 [-0.020,0.012]
	-0.0.51 [-0.088,-0.014]	0.008 [-0.051,0.065]	0.026 [-0.096,0.146]	-0.114 [-0.353,0.119]	0.077 [0.040,0.113]	0.039 [-0.060,0.140]	0.100 [0.058,0.141]	-0.079 [-0.127,-0.031]	0.043 [-0.067,0.019]	0.054 [0.027,0.080]	0.033 [0.005,0.061]	-0.020 [-0.060,0.020]
**Context (BP)**	0.096 [-0.037,-0.228]	1.325 [0.732,1.942]	0.834 [-0.129,1.780]	-0.197 [-3.081,2.725]	-0.181 [-0.456,0.088]	-0.200 [-0.687,0.290]	0.182 [-0.327,0.685]	-0.077 [-0.228,0.084]	-0.022 [-0.130,0.172]	-0.036 [-0.192,0.122]	-0.010 [-0.125,0.138]	-0.137 [-0.456,0.193]
	0.067 [-0.074,0.212]	1.272 [0.560,1.980]	0.823 [-0.197,1.1853]	-0.706 [-3.831,2.483]	-0.247 [-0.526,0.040]	-0.199 [-0.741,0.334]	0.016 [-0.533,0.490]	-0.031 [-0.192,0.130]	0.048 [-0.115,0.215]	0.001 [-0.169,0.173]	-0.061 [-0.203,0.081]	-0.206 [-0.551,0.143]
**Sample**	0.142 [0.073,0.210]	0.123 [0.050,0.193]	0.138 [0.070,0.207]	0.140 [0.075,0.206]	0.141 [0.070, 0.213]	0.139 [0.074,0.205]	0.137 [0.071,0.205]	0.140 [0.070,0.205]	0.148 [0.079,0.219]	0.148 [0.083,0.216]	0.132 [0.066,0.198]	0.139 [0.071,0.209]
	0.122 [-0.005,0.195]	0.097 [0.024,0.170]	0.015 [0.040,0.187]	0.018 [0.045,0.191]	0.122 [0.050,0.193]	0.115 [0.043,0.188]	0.115 [0.040,0.188]	0.118 [0.043,0.190]	0.12 [0.048,0.193]	0.116 [0.040,0.193]	0.114 [0.040,0.187]	0.119 [0.047,0.193]
**Social Media Use (WP) × Context (WP)**	0.020 [-0.016,0.055]	-0.121 [-0.178,-0.064]	-0.069 [-0.193,0.054]	-0.131 [-0.395,0.138]	-0.032 [-0.074,0.010]	0.096 [-0.005,0.196]	0.004 [-0.045,0.052]	0.033 [-0.017,0.083]	0.020 [-0.007,0.047]	0.022 [-0.009,0.053]	-0.044 [-0.075,-0.012]	-0.028 [-0.074,0.017]
	0.042 [0.002,0.082]	-0.044 [-0.114,0.026]	-0.068 [-0.221,0.089]	-0.009 [-0.401,0.373]	-0.026 [-0.072,0.019]	-0.092 [-0.240,0.054]	-0.046 [-0.097,0.004]	-0.031 [-0.089,0.028]	0.016 [-0.012,0.044]	-0.035 [-0.068,-0.002]	0.000 [-0.031,0.031]	-0.003 [-0.057,0.051]
**Social Media Use (WP) × Context (BP)**	-0.011 [-0.061,0.039]	-0.053 [-0.269,0.164]	-0.075 [-0.452,0.306]	-0.096 [-1.272,1.105]	-0.039 [-0.141,0.064]	0.029 [-0.170,0.231]	-0.099 [-0.261,0.062]	0.041 [-0.014,0.097]	0.006 [-0.050,0.062]	0.021 [-0.037,0.078]	-0.030 [-0.079,0.018]	-0.017 [-0.141,0.106]
	0.016 [-0.034,0.065]	-0.062 [-0.289,-0.160]	0.148 [-0.273,0.564]	0.622 [-0.723,1.944]	-0.018 [-0.116,0.081]	-0.056 [-0.279,0.165]	-0.065 [-0.222,0.092]	-0.031 [-0.087,0.025]	-0.006 [-0.063,0.051]	-0.013 [-0.073,0.046]	0.011 [-0.037,0.059]	-0.002 [-0.124,0.120]
**Social Media Use (BP) × Context (BP)**	0.388 [-0.322,1.070]	1.345 [-3.902,1.229]	4.518 [-1.819,10.656]	-1.843 [-18.861,15.135]	-0.092 [-1.624,1.468]	0.839 [-4.222,2.642]	0.182 [-1.729,2.104]	0.013 [-0.768,0.782]	0.097 [-0.687,0.856]	-0.030 [-0.766,0.834]	-0.280 [-0.975,0.417]	0.902 [-0.642,2.478]
	0.127 [-0.065,0.321]	0.367 [-0.673,1.404]	-0.689 [-1.962,0.572]	-0.941 [-6.069,2.200]	-0.761 [-1.198,-0.330]	-0.747 [-1.569,0.074]	-0.767 [-1.551,0.023]	0.066 [-0.151,0.285]	0.192 [-0.026,0.409]	-0.179 [-0.422,0.061]	-0.066 [-0.275,0.141]	-0.640 [-1.133,-0.130]

Note: White rows correspond to the effects of social media use (vs non-use) and grey rows correspond to the effects of social media use duration. 95% Bayesian credible intervals are presented below each co-efficient in square brackets.

Table S39: Context Moderators (Momentary Affect Balance) (Mega-analysis)

	**Home**	**Nature**	**Gym**	**Religion**	**Social**	**Work**	**Transit**	**Study**	**Alone**	**Family Ties**	**Close Ties**	**Distant Ties**
Intercept	0.981 [0.920,1.044]	0.988 [0.926,1.048]	0.987 [0.927,1.046]	0.981 [0.919,1.041]	0.981 [0.922,1.038]	0.979 [0.917,1.038]	0.997 [0.936,1.057]	0.979 [0.919,1.038]	0.985 [0.923,1.044]	0.979 [0.919,1.040]	0.973 [0.915,1.032]	0.977 [0.915,1.039]
	0973 [0.902,1.044]	0.981 [0.910,1.053]	0.957 [0.887,1.026]	0.953 [0.884,1.023]	0.952 [0.882,1.022]	0.953 [0.882,1.024]	0.970 [0.898,1.040]	0.955 [0.886,1.026]	0.962 [0.893,1.031]	0.051 [0.879,0.022]	0.949 [0.881,1.019]	0.954 [-0.883,0.026]
Context (WP)	-0.274 [-0.297,-0.251]	0.358 [0.325,0.392]	0.305 [0.246,0.363]	0.056 [-0.093,0.201]	0.362 [0.334,0.391]	-0.045 [-0.096,0.007]	0.180 [0.153,0.207]	-0.057 [-0.091,-0.023]	-0.276 [-0.294,-0.259]	0.149 [0.127,0.170]	0.322 [0.299,0.345]	0.077 [0.053,0.101]
	-0.192 [-0.241,-0.143]	0.194 [0.113,0.276]	0.033 [-0.110,0.179]	-0.077 [-0.395,0.243]	0.266 [0.212,0.318]	-0.080 [-0.214,0.055]	0.139 [0.080,0.199]	0.002 [-0.062,0.065]	-0.181 [-0.216,-0.147]	0.099 [0.061,0.136]	0.210 [-0.169,0.251]	-0.045 [-0.015,0.106]
Context (BP)	-0.144 [-0.318,0.042]	2.315 [1.489,3.172]	2.264 [1.039,3.480]	2.994 [-0.908,6.828]	0.527 [0.173,0.889]	0.187 [-0.482,0.846]	1.611 [0.938,2.289]	0.016 [-0.192,0.222]	-0.909 [-1.101,-0.711]	0.000 [-0.218,0.213]	0.720 [0.547,0.897]	0.396 [-0.018,0.811]
	-0.225 [-0.415,-0.033]	2.356 [1.390,3.331]	2.331 [0.951,3.704]	2.982 [-1.247,7.180]	0.516 [0.136,0.895]	0.233 [-0.485,0.955]	0.997 [0.323,1.684]	0.013 [-0.201,0.224]	-0.798 [-1.014,-0.582]	0.033 [-0.267,0.198]	0.652 [0.468,0.836]	0.411 [-0.057,0.875]
Sample	0.167 [0.076,0.258]	0.156 [0.067,0.249]	0.174 [0.085,0.262]	0.179 [0.089,0.268]	0.174 [0.087,0.261]	0.182 [0.094,0.272]	0.145 [0.055,0.237]	0.182 [0.094,0.269]	0.123 [0.032,0.213]	0.182 [0.090,0.271]	0.178 [0.092,0.264]	0.180 [0.088,0.270]
	0.145 [0.046,0.243]	0.119 [0.023,0.218]	0.148 [0.051,0.246]	0.156 [0.058,0.253]	0.159 [0.059,0.255]	0.155 [0.054,0.254]	0.128 [0.031,0.229]	0.154 [0.058,0.252]	0.113 [0.017,0.210]	0.162 [0.063,0.262]	0.167 [0.071,0.262]	0.151 [0.051,0.248]
Social Media Use (WP) × Context (WP)	0.054 [0.003,0.104]	-0.101 [-0.182,-0.021]	-0.206 [-0.364,-0.046]	0.008 [-0.376,0.396]	-0.093 [-0.154,-0.031]	0.022 [-0.113,0.157]	-0.048 [-0.116,0.020]	0.083 [0.012,0.154]	0.111 [0.073,0.149]	-0.041 [-0.084,0.003]	-0.097 [-0.141,-0.052]	-0.056 [-0.118,0.006]
	0.070 [0.016,0.124]	-0.123 [-0.227,-0.021]	-0.196 [-0.377,-0.011]	-0.290 [-0.781,0.185]	-0.063 [-0.132,0.005]	0.093 [-0.253,0.065]	-0.050 [-0.126,0.024]	-0.022 [-0.100,0.057]	0.023 [-0.017,0.063]	0.005 [-0.041,0.050]	-0.015 [-0.061,0.032]	-0.046 [-0.116,0.026]
Social Media Use (WP) × Context (BP)	0.050 [-0.023,0.123]	-0.307 [-0.622,0.010]	0.091 [-0.456,0.635]	-0.371 [-2.118,1.367]	-0.128 [-0.280,0.024]	-0.049 [-0.345,0.245]	-0.278 [-0.515,0.038]	0.050 [-0.036,0.136]	0.071 [-0.010,0.152]	-0.000 [-0.085,0.084]	-0.047 [-0.119,0.025]	-0.083 [-0.268,0.099]
	0.0123 [-0.054,0.079]	-0.128 [-0.426,0.164]	-0.254 [-0.801,0.292]	-0.478 [-2.209,1.262]	-0.049 [-0.181,0.085]	-0.065 [-0.346,-0.220]	-0.076 [-0.284,0.129]	-0.039 [-0.115,0.035]	0.047 [-0.028,0.121]	0.022 [-0.055,0.098]	-0.058 [-0.122,0.007]	-0.038 [-0.202,0.127]
Social Media Use (BP) × Context (BP)	-0.170 [-0.814,0.117]	0.476 [-2.889,3.854]	8.173 [-0.018,16.385]	-9.698 [-32.229,12.450]	0.162 [-1.795,2.152]	-2.125 [-6.707,2.386]	-0.522 [-3.056,2.078]	0.138 [-0.891,1.184]	-0.405 [-1.442,0.617]	-0.224 [-1.292,0.844]	0.016 [-0.869,0.916]	1.546 [-0.494,3.612]
	-0.196 [-0.067,0.468]	1.196 [-0.199,2.602]	-0.213 [-1.907,1.506]	1.104 [-4.458,6.688]	-0.768 [-1.341,-0.174]	-0.633 [1.711,0.468]	0.069 [-0.984,1.141]	0.037 [-0.266,0.340]	0.080 [-0.211,0.372]	0.035 [-0.288,0.349]	0.033 [-0.242,0.309]	-0.547 [-1.206,0.119]

Note: White rows correspond to the effects of social media use (vs non-use) and grey rows correspond to the effects of social media use duration. 95% Bayesian credible intervals are presented below each co-efficient in square brackets.

Table S40: Context Moderators (Momentary Feelings of Being Accepted) (Mega-analysis)

	Home	Nature	Gym	Religion	Social	Work	Transit	Study	Alone	Family Ties	Close Ties	Distant Ties
**Intercept**	2.542<br>[2.488,2.596]	2.547<br>[2.494,2.601]	2.539<br>[2.485,2.594]	2.537<br>[2.483,2.593]	2.540<br>[2.489,2.591]	2.533<br>[2.481,2.586]	2.544<br>[2.490,2.597]	2.535<br>[2.478,2.591]	2.555<br>[2.51,2.596]	2.544<br>[2.490,2.599]	2.533<br>[2.483,2.585]	2.531<br>[2.477,2.585]
	2.525<br>[2.466,2.584]	2.527<br>[2.466,2.587]	2.510<br>[2.453,2.569]	2.510<br>[2.452,2.570]	2.509<br>[2.450,2.567]	2.506<br>[2.447,2.566]	2.524<br>[2.463,2.585]	2.509<br>[2.451,2.569]	2.520<br>[2.463,2.577]	2.510<br>[2.449,2.571]	2.292<br>[2.433,2.551]	2.508<br>[2.449,2.568]
**Context (WP)**	-0.196<br>[-0.213,-0.179]	0.190<br>[0.165,0.216]	0.103<br>[0.066,0.142]	0.141<br>[0.031,0.249]	0.270<br>[0.248,0.292]	0.045<br>[0.002,0.088]	0.137<br>[0.117,0.158]	-0.043<br>[-0.068,-0.018]	-0.266<br>[-0.282,-0.25]	0.163<br>[0.145,0.181]	0.284<br>[0.264,0.304]	0.050<br>[0.032,0.068]
	-0.137<br>[-0.174,-0.101]	0.100<br>[0.043,0.156]	0.056<br>[-0.045,0.157]	0.110<br>[-0.147,0.367]	0.189<br>[0.147,0.231]	0.003<br>[-0.091,0.097]	0.125<br>[0.084,0.166]	-0.004<br>[-0.053,0.046]	-0.212<br>[-0.239,-0.185]	0.126<br>[0.097,0.156]	0.222<br>[-0.190,0.254]	-0.049<br>[-0.09,0.089]
**Context (BP)**	-0.235<br>[-0.391,-0.081]	1.981<br>[1.218,2.707]	1.993<br>[0.876,3.105]	2.430<br>[-1.006,5.969]	0.700<br>[0.389,1.022]	-0.067<br>[-0.645,0.559]	1.148<br>[0.561,1.767]	0.153<br>[-0.034,0.340]	-1.053<br>[-1.215,-0.89]	0.039<br>[-0.146,0.224]	0.731<br>[0.580,0.882]	0.527<br>[0.156,0.909]
	-0.341<br>[-0.505,-0.177]	2.138<br>[1.292,2.978]	1.928<br>[0.746,3.111]	1.620<br>[-1.997,5.334]	0.843<br>[0.515,1.158]	-0.114<br>[-0.726,0.499]	0.912<br>[0.303,1.506]	0.184<br>[-0.004,0.374]	-0.965<br>[-1.143,-0.785]	0.030<br>[-0.232,0.170]	0.756<br>[0.598,0.917]	0.469<br>[0.059,0.873]
**Sample**	0.066<br>[-0.012,0.144]	0.057<br>[-0.022,0.142]	0.088<br>[0.005,0.167]	0.090<br>[0.010,0.176]	0.077<br>[0.002,0.152]	0.102<br>[0.026,0.181]	0.067<br>[-0.012,0.146]	0.094<br>[0.004,0.178]	0.023<br>[-0.053,0.100]	0.073<br>[-0.007,0.151]	0.086<br>[0.010,0.162]	0.097<br>[0.021,0.177]
	0.060<br>[-0.024,0.147]	0.049<br>[-0.034,0.134]	0.077<br>[-0.007,0.162]	0.082<br>[-0.004,0.165]	0.075<br>[-0.009,0.157]	0.085<br>[-0.002,0.172]	0.061<br>[-0.025,0.145]	0.083<br>[-0.003,0.167]	0.028<br>[-0.053,0.110]	0.085<br>[-0.005,0.172]	0.098<br>[0.014,0.180]	0.082<br>[-0.002,0.166]
**Social Media Use (WP) × Context (WP)**	0.039<br>[-0.003,0.076]	-0.068<br>[-0.125,-0.010]	-0.047<br>[-0.157,0.063]	0.041<br>[-0.239,0.326]	-0.087<br>[-0.134,-0.040]	0.067<br>[-0.030,0.163]	-0.005<br>[-0.052,0.041]	0.075<br>[0.025,0.125]	0.072<br>[0.046,0.097]	-0.033<br>[-0.065,-0.002]	-0.047<br>[-0.076,-0.017]	0.002<br>[-0.041,0.045]
	0.028<br>[-0.013,0.067]	-0.041<br>[-0.117,0.034]	0.004<br>[-0.128,0.135]	0.108<br>[-0.416,0.630]	-0.028<br>[-0.079,0.022]	-0.167<br>[-0.290,-0.045]	-0.033<br>[-0.084,0.019]	-0.015<br>[-0.069,0.040]	0.025<br>[-0.005,0.055]	0.017<br>[-0.015,0.050]	-0.030<br>[-0.065,0.006]	-0.007<br>[-0.056,0.043]
**Social Media Use (WP) × Context (BP)**	0.006<br>[-0.042,0.055]	-0.127<br>[-0.341,0.091]	0.106<br>[-0.265,0.478]	-1.357<br>[-2.548,-0.187]	-0.057<br>[-0.158,0.044]	0.043<br>[-0.156,0.243]	-0.172<br>[-0.334,-0.010]	0.046<br>[-0.012,0.102]	0.074<br>[0.022,0.128]	-0.027<br>[-0.083,0.030]	-0.033<br>[-0.079,0.015]	-0.073<br>[-0.193,0.050]
	0.008<br>[-0.040,0.058]	-0.177<br>[-0.394,0.039]	0.072<br>[-0.334,0.479]	1.174<br>[-0.213,2.570]	-0.099<br>[-0.194,-0.002]	-0.218<br>[-0.435,-0.007]	-0.199<br>[-0.353,-0.044]	0.036<br>[-0.021,0.093]	0.057<br>[0.002,0.110]	0.015<br>[-0.043,0.073]	-0.050<br>[-0.097,-0.002]	-0.048<br>[-0.174,0.078]
**Social Media Use (BP) × Context (BP)**	0.153<br>[-0.006,0.734]	1.276<br>[-0.1679,4.123]	7.192<br>[0.125,14.513]	-5.194<br>[-25.200,15.145]	1.141<br>[-0.554,2.884]	-1.073<br>[-5.168,2.876]	0.747<br>[-1.376,2.846]	0.111<br>[-0.773,1.014]	-0.474<br>[-1.374,0.401]	-0.617<br>[-1.577,0.354]	0.207<br>[-0.522,0.938]	1.692<br>[-0.083,3.484]
	-0.043<br>[-0.183,0.267]	0.585<br>[-0.622,1.792]	0.870<br>[-0.623,2.376]	1.785<br>[-2.966,6.498]	-0.470<br>[-0.959,0.019]	0.191<br>[-0.781,1.155]	0.161<br>[-0.776,1.081]	0.091<br>[-0.172,0.351]	0.105<br>[-0.132,0.348]	0.264<br>[-0.003,0.537]	-0.113<br>[-0.348,0.128]	-0.107<br>[-0.703,0.477]

Note: White rows correspond to the effects of social media use (vs non-use) and grey rows correspond to the effects of social media use duration. 95% Bayesian credible intervals are presented below each co-efficient in square bracket

Table S41: Wellbeing Comparisons between Exploratory and Confirmatory Samples

	**Lonely**			**Accepted**			**Affect Balance**			**Stress**		
Predictors	*Estimates*	*CI*	*p*	*Estimates*	*CI*	*p*	*Estimates*	*CI*	*p*	*Estimates*	*CI*	*p*
Intercept	3.43	3.39 – 3.47	**<0.001**	2.54	2.49 – 2.59	**<0.001**	0.98	0.92 – 1.04	**<0.001**	2.82	2.78 – 2.87	**<0.001**
Spring 2021 Sample (Relative to Fall 2020 Sample)	0.01	-0.05 – 0.07	0.646	0.10	0.02 – 0.18	**0.012**	0.17	0.08 – 0.26	**<0.001**	0.12	0.06 – 0.19	**<0.001**
**Random Effects**												
σ^2	0.35			0.46			1.00			0.56		
τ_{00}	0.34 $_{id}$			0.58 $_{id}$			0.74 $_{id}$			0.43 $_{id}$		
ICC	0.49			0.56			0.42			0.43		
N	1502 $_{id}$			1502 $_{id}$			1502 $_{id}$			1502 $_{id}$		
Observations	116200			112236			116014			116320		
Marginal R^2 / Conditional R^2	0.000 / 0.491			0.002 / 0.563			0.004 / 0.426			0.004 / 0.435		

Figure S3: Proportion of Time Spent in Different Contexts Across Samples

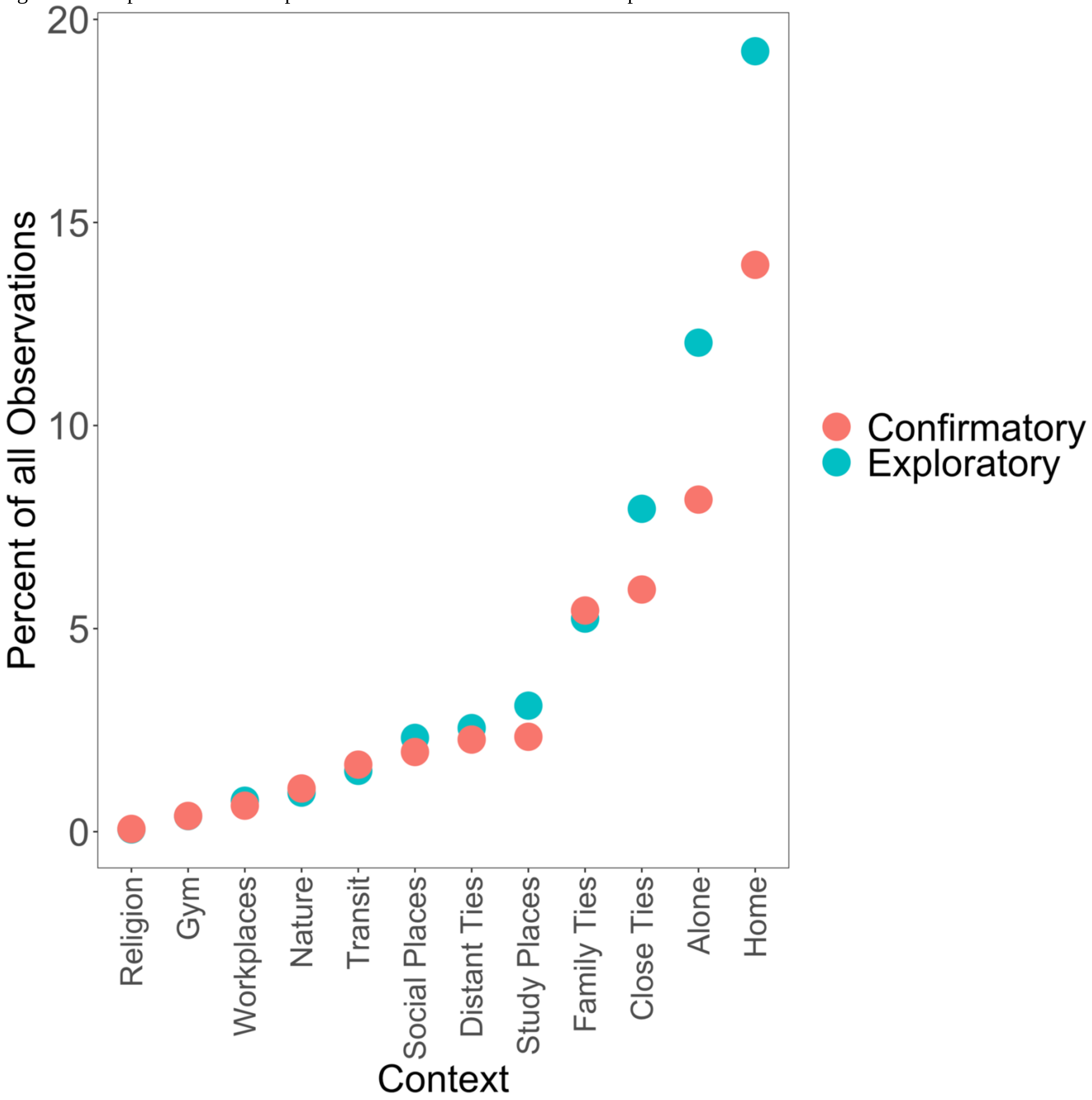

Figure S4: Social Media Use By Context Across Samples

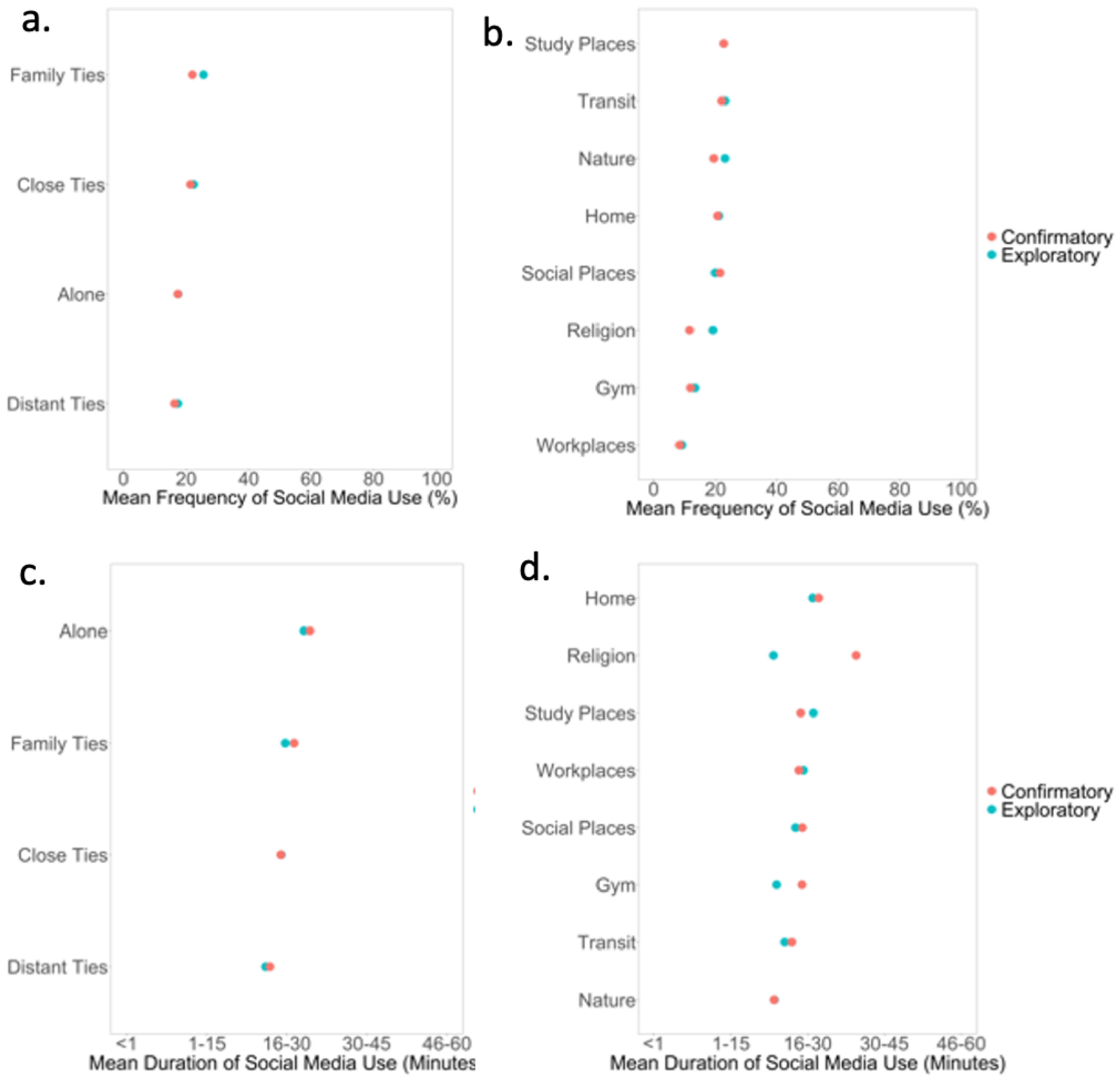

Table S42: Details of Measured Variables

Name of Variable	Tye of Variable	Response Type	Responses & Coding
**Repeated Measures**			
Social Media Use (vs Non-Use)	Independent	Categorical	0 = Non-Social Media Use Activities<br>1= Social Media Use
Momentary Social Media Use Duration<br>("in the past hour")	Independent	Ordinal	1=1-15 minutes.<br>2=16-30 minutes.<br>3 =31-45 minutes.<br>4 =46-60 minutes
Momentary Wellbeing ("right now")	Dependent	Numerical	1. Repeated measures data was collected from the following 7 momentary scales:<br>a. Angry (Reverse-Scored)<br>b. Worried (Reverse-Scored)<br>c. Happy<br>d. Sad (Reverse-Scored)<br>e. Stressed (Reverse-Scored)<br>f. Lonely (Reverse-Scored)<br>g. Accepted<br>2. Affect balance(Schimmack, 2009), computed through the following formula:<br>$Affect\ Balance_{ti} = Happiness_{ti} - \frac{(Anger_{ti} + Worry_{ti} + Sadness_{ti})}{3}$<br>3. Momentary Loneliness<br>4. Momentary Stress<br>5. Momentary Feelings of Being Accepted
Momentary Physical Context<br>("in the past hour")	Moderator	Categorical	0 = All other categories
Social			1 = Bar/Party OR Café/Restaurant OR Friend's House, Fraternity/Sorority House
Nature			1 = Outdoor Park/in-nature
Transit			1= Vehicle
Work			1= Work
Gym			1 = Gym
Study			1 = University Campus OR Library
Religion			1 = Religion
Momentary Social Context	Moderator	Categorical	0 = All other categories
Distant Ties Interaction Partner			1 = Classmates/Students OR Co-Workers OR Strangers
Close Ties Interaction Partner			1= Friends OR Roommates OR Significant Others
Family Ties Interaction Partner			1= Family
**Cross-Sectional Measures**			
Demographics			
Sex	Control	Binary	
Age	Control	Numerical	Continuous Number between 18-25

Big Five Traits (Soto & John, 2017)			
Openness	Moderator	Numerical	Continuous Number between 1-5
Conscientiousness	Moderator	Numerical	Continuous Number between 1-5
Extraversion	Moderator	Numerical	Continuous Number between 1-5
Agreeableness	Moderator	Numerical	Continuous Number between 1-5
Neuroticism	Moderator	Numerical	Continuous Number between 1-5
Wellbeing Traits			
Social Connectedness (Hughes et al., 2004)	Moderator	Numerical	Continuous Number between 1-5
Depression (Radloff, 1977)	Moderator	Numerical	Continuous Number between 1-5
Trait Affect Balance (Richter et al., 2017)	Moderator	Numerical	These dispositional tendencies were aggregated:<br>1. Anger<br>2. Worry<br>3. Happy<br>4. Sadness<br>5. Enthusiastic<br>6. Relaxed<br>Formula for aggregation:<br>$Affect\ Balance_i = Happiness_i - \frac{(Anger_i + Worry_i + Sadness_i)}{3}$
Satisfaction With Life (Diener et al., 1985)	Moderator	Numerical	Continuous Number between 1-7
Loneliness (Luhmann et al., 2016)	Moderator	Numerical	Continuous Number between 1-4
